# Supplementary material for: Development of a multi-epitope vaccine against Acinetobacter baumannii: A comprehensive approach to combating antimicrobial resistance
Source: PLoS One. 2025 Mar 10;20(3):e0319191. doi: 10.1371/journal.pone.0319191 (PMC11892874; doi:10.1371/journal.pone.0319191)
Supplement: S1 Table — (DOCX) [file pone.0319191.s001.docx]

**Supplementary Table 1:** Characterization of 138 extracellular and outer membrane proteins of *A. baumannii* strain VB7036

| No | Accession number | Subcellular localization (Psortb)/Score | Transmembrane (TMHMM - 2.0) | AGNicity score (Vaxigen) | Alergenecity | Human Blast | VICMpred | EGGNOG | CD search | prevalence |
| --- | --- | --- | --- | --- | --- | --- | --- | --- | --- | --- |
| 1 | CP050523_CDS_HBM81_00635 | OMP/9.93 | outside | 0.8110 (AGN) | Allergen | - | Cellular process | M Cell wall/membrane/envelope biogenesis nucleoside-specific channel forming porin activity | Tsx superfamily. 72.23% | |
| 2 | CP050523_CDS_HBM81_00710 | OMP/9.99 | outside | 0.6387 (AGN) | Non-Allergen | - | Cellular process | P Inorganic ion transport and metabolism siderophore transport | OM_channels superfamily | 76.39% |
| 3 | CP050523_CDS_HBM81_00765 | OMP/9.93 | outside | 0.6587 (AGN) | Non-Allergen | - | Virulence factors | M Cell wall/membrane/envelope biogenesis heme binding | Peptidase_M23 superfamily | 93.90% |
| 4 | CP050523_CDS_HBM81_00790 | OMP/10.00 | outside | 0.6204 (AGN) | Non-Allergen | - | Cellular process | P Inorganic ion transport and metabolism siderophore transport | PRK10044 superfamily | 94.16% |
| 5 | CP050523_CDS_HBM81_00825 | OMP/ 9.52 | outside | 0.8157 (AGN) | Allergen | - | Cellular process | S Function unknown Putative general bacterial porin | Porin_7 superfamily | 100.00% |
| 6 | CP050523_CDS_omp33-36 | OMP/9.52 | outside | 0.7580 (AGN) | Allergen | - | Metabolism Molecule | S Function unknown Putative general bacterial porin | Porin_7 superfamily | 94.92% |
| 7 | CP050523_CDS_HBM81_01100 | OMP/ 9.72 | outside | 0.6869 (AGN) | Non-Allergen | + | Cellular process | S Function unknown beta-lactamase activity | TPR superfamily | 94.92% |
| 8 | CP050523_CDS_HBM81_01150 | Extracellular/ 9.72 | outside | 0.4361 (AGN) | Non-Allergen | + | cellular Process | F Nucleotide transport and metabolism neuron death in response to oxidative stress | NUC1 superfamily | 77.41% |
| 9 | CP050523_CDS_HBM81_01340 | OMP/10.00 | outside | 0.5611 (AGN) | Non-Allergen | - | Cellular process | MU Cell wall/membrane/envelope biogenesis, Intracellular trafficking, secretion, and vesicular transport Outer membrane efflux protein | TolC superfamily | 93.65% |
| 10 | CP050523_CDS_HBM81_01480 | OMP/10.00 | outside | 0.6662 (AGN) | Non-Allergen | - | Cellular process | U Intracellular trafficking, secretion, and vesicular transport Type ii and iii secretion system protein | IV_pilus_PilQ superfamily | 93.40% |
| 11 | CP050523_CDS_HBM81_01635 | Extracellular/ 9.64 | outside | 0.6197 (AGN) | Allergen | - | Cellular process | NU Cell motility, Intracellular trafficking, secretion, and vesicular transport Tfp pilus assembly protein tip-associated adhesin | PilY1 superfamily | 100.00% |
| 12 | CP050523_CDS_HBM81_01830 | Extracellular/10 | outside | 0.5522 (AGN) | Allergen | + | Metabolism Molecule | O Post-translational modification, protein turnover, and chaperones Belongs to the peptidase S8 family | Peptidases_S8_S53 superfamily | 100.00% |
| 13 | CP050523_CDS_HBM81_01835 | Extracellular/ 9.65 | outside | 0.4363 (AGN) | Non-Allergen | - | Cellular process | S Function unknown Not Available | ND | 94.67% |
| 14 | CP050523_CDS_HBM81_01970 | Extracellular/9.64 | outside | 0.7338 (AGN) | Non-Allergen | - | Cellular process | Q Secondary metabolites biosynthesis, transport, and catabolism ABC-type transport system involved in resistance to organic solvents, periplasmic component | MlaD superfamily | 26.14% |
| 15 | CP050523_CDS_HBM81_02285 | Extracellular/ 9.96 | outside | 0.5627 ( AGN ) | Allergen | + | Information Molecule | S Function unknown Bacterial collagen, middle region | ND |  |
| 16 | CP050523_CDS_HBM81_02605 | OMP/9.49 | outside | 0.9173 (AGN) | Allergen | - | Virulence factors | S Function unknown Not Available | PRK15316 superfamily | 100.00% |
| 17 | CP050523_CDS_HBM81_02800 | OMP/10 | outside | 0.7222 (AGN) | Allergen | - | Cellular process | P Inorganic ion transport and metabolism TonB-dependent receptor | Zn_piracy_ZnuD superfamily | 100.00% |
| 18 | CP050523_CDS_HBM81_02885 | OMP/10.00 | outside | 0.6510 (AGN) | Allergen | - | Metabolism Molecule | P Inorganic ion transport and metabolism TonB-dependent receptor | BtuB superfamily | ND |
| 19 | CP050523_CDS_HBM81_03020 | OMP/9.93 | outside | 0.5497 (AGN) | Allergen | - | Metabolism Molecule | M Cell wall/membrane/envelope biogenesis wide pore channel activity | OprB superfamily | 100.00% |
| 20 | CP050523_CDS_HBM81_03060 | OMP/10.00 | outside | 0.8481 (AGN) | Non-Allergen | - | Cellular process | M Cell wall/membrane/envelope biogenesis chlorophyll binding | OmpA_C-like superfamily | 94.41% |
| 21 | CP050523_CDS_HBM81_03120 | Extracellular/ 9.65 | outside | 0.7865 (AGN) | Allergen | - | Information Molecule | P Inorganic ion transport and metabolism TonB-dependent receptor | Plug superfamily | |
| 22 | CP050523_CDS_HBM81_03330 | Extracellular/ 8.89 | outside | 0.6518 (AGN) | Non-Allergen | + | Metabolism Molecule | M Cell wall/membrane/envelope biogenesis Esterase of the alpha-beta hydrolase superfamily | Patatin_and_cPLA2 superfamily | 100.00% |
| 23 | CP050523_CDS_HBM81_03535 | OMP/ 9.52 | outside | 0.7928 (AGN) | Allergen | - | Cellular process | U Intracellular trafficking, secretion, and vesicular transport Involved in a type II secretion system (T2SS, formerly general secretion pathway, GSP) for the export of proteins | DcaP superfamily | 100.00% |
| 24 | CP050523_CDS_adeK | OMP/10.00 | outside | 0.6450 (AGN) | Non-Allergen | - | Cellular process | M Cell wall/membrane/envelope biogenesis RND efflux system, outer membrane lipopro | efflux_OM_AdeK superfamily | 94.92% |
| 25 | CP050523_CDS_HBM81_03930 | Extracellular/ 9.72 | outside | 0.4901 (AGN) | Non-Allergen | - | Metabolism Molecule | P Inorganic ion transport and metabolism PhoD-like phosphatase | MPP_superfamily superfamily | 94.67% |
| 26 | CP050523_CDS_HBM81_04230 | OMP/9.52 | outside | 0.5135 (AGN) | Allergen | - | Cellular process | MU Cell wall/membrane/envelope biogenesis, Intracellular trafficking, secretion, and vesicular transport Outer membrane efflux protein | TolC superfamily | 20.30% |
| 27 | CP050523_CDS_HBM81_04315 | Extracellular/ 9.65 | outside | 0.6298 (AGN) | Allergen | - | Virulence factors | S Function unknown Not Available | CSLREA_Nterm superfamily | 100.00% |
| 28 | CP050523_CDS_pal | OMP/10.00 | outside | 0.8113 (AGN) | Non-Allergen | - | Metabolism Molecule | M Cell wall/membrane/envelope biogenesis chlorophyll binding | OmpA_C-like superfamily | 93.90% |
| 29 | CP050523_CDS_HBM81_04630 | OMP/9.93 | outside | 0.3123 (Probable NON-AGN ). | Non-Allergen | + | Metabolism Molecule | M Cell wall/membrane/envelope biogenesis lipid binding | lipocalin_FABP superfamily | ND |
| 30 | CP050523_CDS_HBM81_05435 | OMP/9.49 | outside | 0.5920 (AGN) | Allergen | - | Virulence factors | O Post-translational modification, protein turnover, and chaperones Phage-related protein, tail | ND | 70.55% |
| 31 | CP050523_CDS_HBM81_05495 | OMP/9.52 | outside | 0.4587 (AGN) | Non-Allergen | + | Cellular process | L Replication, recombination and repair Peptidase, M16 | PqqL superfamily | 41.11% |
| 32 | CP050523_CDS_HBM81_05635 | Extracellular/ 9.64 | outside | 0.7264 (AGN) | Allergen | - | Virulence factors | S Function unknown LPXTG cell wall anchor motif M Cell wall/membrane/envelope biogenesis Domain of unknown function | Ig_like_BLP2 superfamily | 93.90% |
| 33 | CP050523_CDS_HBM81_05695 | OMP/ 9.92 | outside | 0.6465 (AGN) | Non-Allergen | - | Metabolism Molecule | D Cell cycle control, cell division, chromosome partitioning peptidase | nlpD superfamily | 92.63% |
| 34 | CP050523_CDS_HBM81_05855 | OMP/9.52 | outside | 0.5477 (AGN) | Allergen | - | Virulence factors | P Inorganic ion transport and metabolism Not Available | ND | 89.59% |
| 35 | CP050523_CDS_HBM81_05965 | OMP/10.00 | outside | 0.5532 (AGN) | Non-Allergen | - | Metabolism Molecule | P Inorganic ion transport and metabolism siderophore transport | OM_channels superfamily | 94.67% |
| 36 | CP050523_CDS_HBM81_06095 | OMP/10.00 | outside | 0.5679 (AGN) | Allergen | - | Virulence factors | P Inorganic ion transport and metabolism siderophore transport | OM_channels superfamily | 90.10% |
| 37 | CP050523_CDS_HBM81_06435 | OMP/ 9.49 | outside | 0.5857 (AGN) | Allergen | - | Cellular process | S Function unknown Not Available O Post-translational modification, protein turnover, and chaperones Phage-related protein, tail | ND | ND |
| 38 | CP050523_CDS_adeH | OMP/10.00 | outside | 0.5295 (AGN) | Non-Allergen | - | Cellular process | M Cell wall/membrane/envelope biogenesis RND efflux system, outer membrane lipoprote | outer_NodT superfamily | 75.10% |
| 39 | CP050523_CDS_tsaD | Extracellular/9.71 | outside | 0.5130 (AGN) | Non-Allergen | + | Cellular process | J Translation, ribosomal structure and biogenesis N(6)-L-threonylcarbamoyladenine synthase activity | PRK09604 superfamily | 93.40% |
| 40 | CP050523_CDS_csuB | Extracellular/9.64 | outside | 0.5742 (AGN) | Non-Allergen | - | Cellular process | S Function unknown Spore Coat Protein U domain | SCPU superfamily | 93.14% |
| 41 | CP050523_CDS_csuD | OMP/10.00 | outside | 0.5258 (AGN) | Non-Allergen | - | Virulence factors | Eukaryota | FimD superfamily | 93.40% |
| 42 | CP050523_CDS_HBM81_07630 | OMP/10.00 | outside | 0.5641 (AGN) | Allergen | - | Virulence factors | M Cell wall/membrane/envelope biogenesis surface antigen | TamA superfamily | 94.16% |
| 43 | CP050523_CDS_HBM81_07880 | OMP/10.00 | outside | 0.5887 (AGN) | Allergen | - | Metabolism Molecule | P Inorganic ion transport and metabolism TonB-dependent receptor | OM_channels superfamily | 93.65% |
| 44 | CP050523_CDS_HBM81_07920 | OMP/10.00 | outside | 0.5768 (AGN) | Non-Allergen | - | Cellular process | P Inorganic ion transport and metabolism Receptor | FhuE superfamily | 100.00% |
| 45 | CP050523_CDS_HBM81_08045 | Extracellular/10.00 | outside | 0.4082 (AGN) | Non-Allergen | - | Metabolism Molecule | K Transcription nuclear-transcribed mRNA catabolic process, deadenylation-dependent decay | PC_PLC superfamily | 88.80% |
| 46 | CP050523_CDS_bamA | OMP/10.00 | outside | 0.6315 (AGN) | Allergen | + | Virulence factors | M Cell wall/membrane/envelope biogenesis membrane organization | OM_YaeT superfamily | 94.67% |
| 47 | CP050523_CDS_HBM81_08365 | Extracellular/9.64 | outside | 0.6897 (AGN) | Allergen | - | Virulence factors | S Function unknown Outer membrane lipoprotein S Function unknown Not Available | YbgF superfamily | 94.92% |
| 48 | CP050523_CDS_HBM81_08435 | Extracellular/10.00 | outside | 0.6377 (AGN) | Allergen | + | Virulence factors | O Post-translational modification, protein turnover, and chaperones Belongs to the peptidase S8 family | Peptidases_S8_S53 superfamily | 2.03% |
| 49 | CP050523_CDS_HBM81_08630 | OMP/ 10.00 | outside | 0.5950 (AGN) | Non-Allergen | - | Virulence factors | P Inorganic ion transport and metabolism siderophore transport | OM_channels superfamily | 59.89% |
| 50 | CP050523_CDS_HBM81_08640 | OMP/ 9.93 | outside | 0.5245 (AGN) | Non-Allergen | - | Metabolism Molecule | M Cell wall/membrane/envelope biogenesis 1-acyl-2-lysophosphatidylserine acylhydrolase activity | OMPLA superfamily | 59.89% |
| 51 | CP050523_CDS_HBM81_08670 | Extracellular/9.64 | outside | 0.5455 (AGN) | Non-Allergen | - | Virulence factors | S Function unknown Not Available | ND | 94.41% |
| 52 | CP050523_CDS_HBM81_08830 | OMP//10.00 | outside | 0.6025 (AGN) | Allergen | - | Cellular process | M Cell wall/membrane/envelope biogenesis wide pore channel activity | OprB superfamily | 100.00% |
| 53 | CP050523_CDS_HBM81_08890 | OMP/9.93 | outside | 0.5282 (AGN) | Allergen | - | Metabolism Molecule | M Cell wall/membrane/envelope biogenesis wide pore channel activity | OprD superfamily | 100.00% |
| 54 | CP050523_CDS_HBM81_09220 | OMP/10.00 | outside | 0.4944 (AGN) | Non-Allergen | + | Information Molecule | O Post-translational modification, protein turnover, and chaperones toxin metabolic process | FhaC superfamily | 100.00% |
| 55 | CP050523_CDS_HBM81_09225 | OMP/ 9.93 | outside | 0.6709 ( AGN ) | Non-Allergen | - | Information Molecule | D Cell cycle control, cell division, chromosome partitioning Cell division factor that enhances FtsZ-ring assembly. Directly interacts with FtsZ and promotes bundling of FtsZ protofilaments, with a reduction in FtsZ GTPase activity | Haemagg_act superfamily | |
| 56 | CP050523_CDS_HBM81_09305 | OMP/9.49 | outside | 0.6789 (AGN) | Allergen | - | Virulence factors | U Intracellular trafficking, secretion, and vesicular transport Involved in a type II secretion system (T2SS, formerly general secretion pathway, GSP) for the export of proteins | DcaP superfamily | 100.00% |
| 57 | CP050523_CDS_HBM81_09315 | Extracellular/10.00 | outside | 0.6264 (AGN) | Allergen | - | Information Molecule | NU Cell motility, Intracellular trafficking, secretion, and vesicular transport translation initiation factor activity | Ig_like_BLP2 superfamily | 100.00% |
| 58 | CP050523_CDS_HBM81_09525 | OMP/10.00 | outside | 0.5442 (AGN) | Non-Allergen | - | Cellular process | MU Cell wall/membrane/envelope biogenesis, Intracellular trafficking, secretion, and vesicular transport type I secretion outer membrane protein, TolC | tolC superfamily | 100.00% |
| 59 | CP050523_CDS_HBM81_09535 | Extracellular/9.65 | outside | 0.6221 (AGN) | Non-Allergen | - | Metabolism Molecule | P Inorganic ion transport and metabolism Nucleotidase that shows phosphatase activity on nucleoside 5'-monophosphates | SurE superfamily | 93.14% |
| 60 | CP050523_CDS_adeC | OMP/10.00 | outside | 0.6142 (AGN) | Non-Allergen | - | Virulence factors | M Cell wall/membrane/envelope biogenesis RND efflux system, outer membrane lipoprotein | TolC superfamily | 92.38% |
| 61 | CP050523_CDS_HBM81_09670 | OMP/9.49 | outside | 0.6269 (AGN) | Allergen | + | Cellular process | Eukaryota | FadL superfamily | 51.52% |
| 62 | CP050523_CDS_HBM81_09680 | Extracellular/ 10.00 | outside | 0.7340 (AGN) | Non-Allergen | - | Virulence factors | NU Cell motility, Intracellular trafficking, secretion, and vesicular transport cell adhesion | FimA superfamily | 100.00% |
| 63 | CP050523_CDS_HBM81_09695 | OMP/10.00 | outside | 0.5733 (AGN) | Non-Allergen | - | Cellular process | Eukaryota | FimD superfamily | 100.00% |
| 64 | CP050523_CDS_HBM81_09700 | Extracellular/ 9.71 | outside | 0.5495 (AGN) | Allergen | - | Virulence factors | NU Cell motility, Intracellular trafficking, secretion, and vesicular transport cell adhesion | Fimbrial superfamily | 100.00% |
| 65 | CP050523_CDS_HBM81_09705 | OMP/9.49 | outside | 0.6357 (AGN) | Non-Allergen | - | Cellular process | S Function unknown Domain of unknown function (DUF4882) | DUF4882 superfamily | ?? |
| 66 | CP050523_CDS_HBM81_09955 | OMP/10.00 | outside | 0.6062 (AGN) | Allergen | - | Cellular process | P Inorganic ion transport and metabolism siderophore transport | OM_channels superfamily | 100.00% |
| 67 | CP050523_CDS_HBM81_10235 | OMP/10.00 | outside | 0.8504 (AGN) | Allergen | - | Cellular process | P Inorganic ion transport and metabolism Not Available | OM_channels superfamily | 94.41% |
| 68 | CP050523_CDS_HBM81_10285 | OMP/10.00 | outside | 0.6476 (AGN) | Non-Allergen | - | Cellular process | P Inorganic ion transport and metabolism Receptor | FhuE superfamily | 100.00% |
| 69 | CP050523_CDS_HBM81_10320 | OMP/9.49 | outside | 0.4691 (AGN) | Allergen | + | Cellular process | S Function unknown Histidine phosphatase superfamily (branch 2) | HP superfamily | 8.12% |
| 70 | CP050523_CDS_HBM81_10370 | OMP/10.00 | outside | 0.5991 (AGN) | Non-Allergen | - | Virulence factors | P Inorganic ion transport and metabolism TonB-dependent receptor | OM_channels superfamily | 56.09% |
| 71 | CP050523_CDS_HBM81_10600 | OMP/10.00 | outside | 0.6381 (AGN) | Allergen | - | Virulence factors | P Inorganic ion transport and metabolism TonB-dependent receptor | OM_channels superfamily | 56.09% |
| 72 | CP050523_CDS_HBM81_10795 | OMP/10.00 | outside | 0.6306 (AGN) | Non-Allergen | - | Virulence factors | M Cell wall/membrane/envelope biogenesis lipopolysaccharide transport | LptD superfamily | 100.00% |
| 73 | CP050523_CDS_HBM81_11010 | Extracellular/ 10.00 | outside | 0.7577 (AGN) | Allergen | - | Virulence factors | NU Cell motility, Intracellular trafficking, secretion, and vesicular transport cell adhesion | FimA superfamily | 3.40% |
| 74 | CP050523_CDS_HBM81_11020 | OMP/10.00 | outside | 0.5391 (AGN) | Non-Allergen | - | Cellular process | Eukaryota | FimD superfamily | 93.65% |
| 75 | CP050523_CDS_HBM81_11025 | Extracellular/ 10.00 | outside | 0.6646 (AGN) | Non-Allergen | - | Virulence factors | NU Cell motility, Intracellular trafficking, secretion, and vesicular transport cell adhesion | Fimbrial superfamily | 90.60% |
| 76 | CP050523_CDS_HBM81_11100 | OMP/9.52 | outside | 0.6991 (AGN) | Allergen | - | Virulence factors | U Intracellular trafficking, secretion, and vesicular transport Involved in a type II secretion system (T2SS, formerly general secretion pathway, GSP) for the export of proteins | DcaP superfamily | 94.16% |
| 77 | CP050523_CDS_HBM81_11775 | Extracellular/ 9.65 | outside | 0.7093 (AGN) | Non-Allergen | - | Cellular process | U Intracellular trafficking, secretion, and vesicular transport Involved in a type II secretion system (T2SS, formerly general secretion pathway, GSP) for the export of proteins | DcaP superfamily | 100.00% |
| 78 | CP050523_CDS_HBM81_11925 | OMP/9.49 | outside | 0.5247 (AGN) | Allergen | - | Cellular process | Q Secondary metabolites biosynthesis, transport, and catabolism PFAM Bacterial OB fold (BOF) protein | ND | 94.92% |
| 79 | CP050523_CDS_HBM81_12270 | Extracellular/ 9.71 | outside | 0.8111 (AGN) | Non-Allergen | - | Metabolism Molecule | S Function unknown Type VI secretion system effector, Hcp | T6SS_HCP superfamily | 94.67% |
| 80 | CP050523_CDS_HBM81_12380 | Extracellular/ 9.71 | outside | 0.6440 (AGN) | Non-Allergen | + | Cellular process | S Function unknown beta-lactamase activity | TPR superfamily | 93.65% |
| 81 | CP050523_CDS_HBM81_12430 | Extracellular/ 9.64 | outside | 0.4238 (AGN) | Non-Allergen | + | Virulence factors | M Cell wall/membrane/envelope biogenesis self proteolysis | RhsA superfamily | 100.00% |
| 82 | CP050523_CDS_HBM81_12565 | OMP/10.00 | outside | 0.5146 (AGN) | Non-Allergen | - | Virulence factors | MU Cell wall/membrane/envelope biogenesis, Intracellular trafficking, secretion, and vesicular transport type I secretion outer membrane protein, TolC | tolC superfamily | 100.00% |
| 83 | CP050523_CDS_HBM81_12795 | OMP/10.00 | outside | 0.5247 (AGN) | Allergen | - | Metabolism Molecule | S Function unknown outer membrane porin, OprD family | OprD superfamily | 94.41% |
| 84 | CP050523_CDS_HBM81_13095 | OMP/10.00 | outside | 0.6953 (AGN) | Non-Allergen | - | Cellular process | P Inorganic ion transport and metabolism siderophore transport | CirA superfamily | 92.63% |
| 85 | CP050523_CDS_HBM81_13125 | OMP/ 9.52 | outside | 0.6471(AGN) | Non-Allergen | + | Information Molecule | M Cell wall/membrane/envelope biogenesis lytic transglycosylase activity | mltD superfamily | 9441.00% |
| 86 | CP050523_CDS_HBM81_13245 | OMP/9.52 | outside | 0.5944 (AGN) | Non-Allergen | - | Cellular process | P Inorganic ion transport and metabolism TonB-dependent receptor | CirA superfamily | 9441.00% |
| 87 | CP050523_CDS_HBM81_13260 | OMP/9.49 | outside | 0.5798 (AGN) | Allergen | - | Virulence factors | S Function unknown Not Available | ND | 94.92% |
| 88 | CP050523_CDS_HBM81_13615 | OMP/9.93 | outside | 0.4538 (AGN) | Allergen | - | Cellular process | M Cell wall/membrane/envelope biogenesis chlorophyll binding | OmpA_C-like superfamily | 94.92% |
| 89 | CP050523_CDS_HBM81_ata | OMP/9.95 | outside | 0.9138 (AGN) | Non-Allergen | - | Information Molecule | UW Intracellular trafficking, secretion, and vesicular transport, Extracellular structures Hep Hag repeat protein | auto_Ata superfamily | 9441.00% |
| 90 | CP050523_CDS_HBM81_13735 | OMP/9.93 | outside | 0.6854 (AGN) | Allergen | - | Metabolism Molecule | MP Cell wall/membrane/envelope biogenesis, Inorganic ion transport and metabolism regulation of cell-substrate adhesion | NlpE superfamily | |
| 91 | CP050523_CDS_HBM81_13790 | OMP/ 9.52 | outside | 0.6150 (AGN) | Allergen | - | Metabolism Molecule | S Function unknown Capsule assembly protein Wzi | Caps_assemb_Wzi superfamily | |
| 92 | CP050523_CDS_HBM81_13880 | OMP/10.00 | outside | 0.8130 (AGN) | Non-Allergen | - | Cellular process | P Inorganic ion transport and metabolism TonB-dependent receptor | PRK13524 superfamily | |
| 93 | CP050523_CDS_HBM81_13990 | OMP/9.52 | outside | 0.7198 (AGN) | Allergen | - | Cellular process | S Function unknown Not Available | ND |  |
| 94 | CP050523_CDS_HBM81_14205 | OMP/9.49 | outside | 0.5105 (AGN) | Non-Allergen | - | Cellular process | O Post-translational modification, protein turnover, and chaperones FOG TPR repeat | OM_channels superfamily | |
| 95 | CP050523_CDS_HBM81_14210 | Extracellular/ 9.65 | outside | 0.5826 (AGN) | Allergen | - | Cellular process | S Function unknown Not Available | TbpB_B_D superfamily | |
| 96 | CP050523_CDS_HBM81_14215 | OMP/10.00 | outside | 0.5705 (AGN) | Allergen | - | Virulence factors | P Inorganic ion transport and metabolism siderophore transport | TonB-hemin superfamily | |
| 97 | CP050523_CDS_HBM81_14480 | Extracellular/ 9.64 | outside | 0.2850 (Probable NON-AGN ) | Non-Allergen | - | Cellular process | S Function unknown Not Available | ND |  |
| 98 | CP050523_CDS_HBM81_14745 | OMP/ 9.92 | outside | 0.4154 (AGN) | Non-Allergen | - | Cellular process | J Translation, ribosomal structure and biogenesis Gram-negative-bacterium-type cell outer membrane assembly | BamE superfamily | |
| 99 | CP050523_CDS_HBM81_14795 | OMP/10.00 | outside | 0.7523 (AGN) | Non-Allergen | - | Metabolism Molecule | M Cell wall/membrane/envelope biogenesis chlorophyll binding | PRK10510 superfamily | |
| 100 | CP050523_CDS_HBM81_14975 | OMP/9.52 | outside | 0.5754 (AGN) | Allergen | - | Metabolism Molecule | P Inorganic ion transport and metabolism Receptor | OM_channels superfamily | |
| 101 | CP050523_CDS_HBM81_15020 | OMP/9.92 | outside | 0.4531 (AGN) | Non-Allergen | - | Metabolism Molecule | S Function unknown cell envelope organization | BamD superfamily | |
| 102 | CP050523_CDS_HBM81_15285 | Extracellular/ 9.65 | outside | 0.7192 (AGN) | Allergen | - | Virulence factors | S Function unknown Not Available | ND |  |
| 103 | CP050523_CDS_HBM81_15595 | Extracellular/ 9.64 | outside | 1.0620 (AGN) | Allergen | - | Virulence factors | S Function unknown Not Available | ND |  |
| 104 | CP050523_CDS_HBM81_15660 | OMP/ 9.52 | outside | 0.5845 (AGN) | Allergen | - | Metabolism Molecule | I Lipid transport and metabolism long-chain fatty acid transporting porin activity | FadL superfamily | |
| 105 | CP050523_CDS_HBM81_15770 | OMP/ 9.93 | outside | 0.5199 (AGN) | Allergen | - | Virulence factors | P Inorganic ion transport and metabolism copper resistance | CopB superfamily | |
| 106 | CP050523_CDS_HBM81_15835 | Extracellular/ 9.64 | outside | 0.6426 (AGN) | Non-Allergen | - | Virulence factors | S Function unknown Not Available | ND |  |
| 107 | CP050523_CDS_HBM81_15855 | OMP/9.49 | outside | 0.8203 (AGN) | Non-Allergen | - | Cellular process | C Energy production and conversion Not Available | ND |  |
| 108 | CP050523_CDS_HBM81_15865 | Extracellular/ 9.65 | outside | 0.9372 (AGN) | Non-Allergen | - | Virulence factors | S Function unknown Not Available | ND |  |
| 109 | CP050523_CDS_HBM81_15935 | OMP/ 9.92 | outside | 0.5205(AGN) | Non-Allergen | - | Cellular process | M Cell wall/membrane/envelope biogenesis Lipoprotein | MlaA superfamily | |
| 110 | CP050523_CDS_HBM81_16370 | OMP/10.00 | outside | 0.6315 (AGN) | Non-Allergen | - | Cellular process | M Cell wall/membrane/envelope biogenesis RND efflux system, outer membrane lipoprotein | outer_NodT superfamily | |
| 111 | CP050523_CDS_pilW | OMP/9.92 | outside | 0.5105 (AGN) | Non-Allergen | + | Cellular process | NU Cell motility, Intracellular trafficking, secretion, and vesicular transport Pilus assembly protein | type_IV_pilW superfamily | |
| 112 | CP050523_CDS_ndk | Extracellular/ 9.45 | outside | 0.3087 (Probable NON-AGN ) | Allergen | + | Metabolism Molecule | F Nucleotide transport and metabolism Major role in the synthesis of nucleoside triphosphates other than ATP. The ATP gamma phosphate is transferred to the NDP beta phosphate via a ping-pong mechanism, using a phosphorylated active-site intermediate | NDPk superfamily | |
| 113 | CP050523_CDS_HBM81_16725 | Extracellular/ 9.64 | outside | 0.4860 (AGN) | Allergen | - | Cellular process |  | DUF839 superfamily | |
| 114 | CP050523_CDS_HBM81_17565 | OMP/9.49 | outside | 0.5665 (AGN) | Non-Allergen | - | Cellular process | O Post-translational modification, protein turnover, and chaperones Phosphatase | | |
| 115 | CP050523_CDS_HBM81_17585 | OMP/9.93 | outside | 0.6121 (AGN) | Allergen | - | Metabolism Molecule | S Function unknown Not Available | ND |  |
| 116 | CP050523_CDS_gspD | OMP/10.00 | outside | 0.5931 (AGN) | Allergen | - | Cellular process | M Cell wall/membrane/envelope biogenesis Not Available | OmpW superfamily | |
| 117 | CP050523_CDS_HBM81_17770 | OMP/10.00 | outside | 0.5693 (AGN) | Non-Allergen | - | Cellular process | NU Cell motility, Intracellular trafficking, secretion, and vesicular transport general secretion pathway protein D | type_II_gspD superfamily | |
| 118 | CP050523_CDS_HBM81_18105 | OMP/9.93 | outside | 0.6662 (AGN) | Allergen | - | Metabolism Molecule | MU Cell wall/membrane/envelope biogenesis, Intracellular trafficking, secretion, and vesicular transport type I secretion outer membrane protein, TolC | tolC superfamily | |
| 119 | CP050523_CDS_HBM81_18265 | OMP/10.00 | outside | 0.6309 (AGN) | Non-Allergen | - | Metabolism Molecule | S Function unknown outer membrane porin, OprD family | OprD superfamily | |
| 120 | CP050523_CDS_HBM81_18865 | OMP/9.49 | outside | 0.5939(AGN) | Non-Allergen | - | Virulence factors | P Inorganic ion transport and metabolism receptor | TonB-copper superfamily | |
| 121 | CP050523_CDS_HBM81_18985 | OMP/10.00 | outside | 0.6269(AGN) | Non-Allergen | - | Metabolism Molecule | S Function unknown Not Available | ND |  |
| 122 | CP050523_CDS_HBM81_19200 | OMP/9.93 | outside | 0.5064(AGN) | Non-Allergen | - | Metabolism Molecule | P Inorganic ion transport and metabolism siderophore transport | OM_channels superfamily | |
| 123 | CP050523_CDS_HBM81_19215 | OMP/9.92 | outside | 0.5811 (AGN) | Allergen | + | Metabolism Molecule | M Cell wall/membrane/envelope biogenesis polysaccharide export | PRK15078 superfamily | |
| 124 | CP050523_CDS_HBM81_19220 | OMP/9.93 | outside | 0.6121 (AGN) | Non-Allergen | + | Metabolism Molecule | O Post-translational modification, protein turnover, and chaperones Peptidyl-prolyl cis-trans | FkpA superfamily | |
| 125 | CP050523_CDS_HBM81_19245 | Extracellular/ 9.72 | outside | 0.4297 (AGN) | Non-Allergen | - | Metabolism Molecule | O Post-translational modification, protein turnover, and chaperones Peptidyl-prolyl cis-trans | FkpA superfamily | |
| 126 | CP050523_CDS_HBM81_19445 | OMP/9.49 | outside | 0.4692 (AGN) | Non-Allergen | + | Cellular process | K Transcription nuclear-transcribed mRNA catabolic process, deadenylation-dependent decay | PC_PLC superfamily | |
| 127 | CP050523_CDS_HBM81_00420 | Extracellular4.44- OMP/ 4.28 | outside | 0.3529 (Probable NON-AGN ) | Non-Allergen | - | Metabolism Molecule | S Function unknown Matrixin | Peptidase_M10 superfamily | |
| 128 | CP050523_CDS_HBM81_03440 | Extracellula/6.26-OMP/3.73 | outside | 0.6575 (AGN) | Allergen | - | Cellular process | S Function unknown Bacterial protein of unknown function (DUF885) | DUF885 superfamily | |
| 129 | CP050523_CDS_HBM81_03520 | OMP/7.00-Extracellular/2.94 | outside | 0.6246 (AGN) | Allergen | + | Metabolism Molecule | I Lipid transport and metabolism long-chain fatty acid transporting porin activ | FadL superfamily | |
| 130 | CP050523_CDS_HBM81_03830 | Extracellular 6.04-OMP/3.60 | outside | 0.7396 (AGN) | Allergen | + | Information Molecule | K Transcription Esterase of the alpha-beta hydrolase superfamily | Patatin_and_cPLA2 superfamily | |
| 131 | CP050523_CDS_HBM81_04035 | Extracellular/5.02-Periplasmic/4.90 | outside | 0.4451 (AGN) | Non-Allergen | - | Virulence factors | MU Cell wall/membrane/envelope biogenesis, Intracellular trafficking, secretion, and vesicular transport HemY domain protein | Ig_like_BLP2 superfamily | |
| 132 | CP050523_CDS_HBM81_04925 | Extracellular/6.26-OMP/3.73 | outside | 0.4930(AGN) | Allergen | - | Information Molecule | M Cell wall/membrane/envelope biogenesis COG0741 Soluble lytic murein transglycosylase and related regulatory proteins (some contain LysM invasin domains) | Lyz-like superfamily | |
| 133 | CP050523_CDS_HBM81_09790 | Extracellular/5.02-Periplasmic/4.90 | Outside | 0.5511 (AGN) | Allergen | + | Virulence factors | D Cell cycle control, cell division, chromosome partitioning Phage-related minor tail protein | TMP_3 superfamily | |
| 134 | CP050523_CDS_HBM81_13285 | Extracellular/6.04-OMP/3.60 | outside | 0.5027 (AGN) | Allergen | - | Information Molecule | F Nucleotide transport and metabolism nucleotide catabolic process | MPP_superfamily superfamily | |
| 135 | CP050523_CDS_gdhA | OMP/4.74- Cytoplasmic/4.28 | outside | 0.4891(AGN) | Allergen | + | Metabolism Molecule | D Cell cycle control, cell division, chromosome partitioning Phage-related minor tail protein | TMP_3 superfamily | |
| 136 | CP050523_CDS_HBM81_14465 | Extracellular/6.04 -OMP/3.60 | outside | 0.5902 (AGN) | Allergen | + | Information Molecule | E Amino acid transport and metabolism glutamate dehydrogenase [NAD(P)+] activity | PRK09414 superfamily | |
| 137 | CP050523_CDS_HBM81_15270 | Extracellular/6.26- OMP/ 3.73 | outside | 0.7508 (AGN) | Allergen | - | Virulence factors | U Intracellular trafficking, secretion, and vesicular transport conjugation | PANDER_like superfamily | |
| 138 | CP050523_CDS_HBM81_15280 | Extracellular/6.26-OMP/ 3.73 | outside | 0.6397 (AGN) | Allergen | - | Virulence factors | S Function unknown Not Available | ND |  |

**Supplementary Table 2. Epitope Shuffling Techniques for Optimal Arrangement with Highest Antigenicity Score**

| **No** | **Arrangement** | **Antigenicity Score** |
| --- | --- | --- |
| 1 | LATQSARDGPGPGLPIFDWGTRRANVKISETDQKIALSDGPGPGSSTINEDPNSGTNNGNLTSGSCTPTTSDNGAEDSTGPGPGKTGDSPYEIGLDELSTGKGGPGPGNSRNSVRYGWKGERDTRGDSNWVPAE | 1.7398 |
| 2 | LATQSARDGPGPGLPIFDWGTRRANVKISETDQKIALSDGPGPGSSTINEDPNSGTNNGNLTSGSCTPTTSDNGAEDSTGPGPGNSRNSVRYGWKGERDTRGDSNWVPAEGPGPGKTGDSPYEIGLDELSTGKG | 1.6926 |
| 3 | LATQSARDGPGPGLPIFDWGTRRANVKISETDQKIALSDGPGPGKTGDSPYEIGLDELSTGKGGPGPGSSTINEDPNSGTNNGNLTSGSCTPTTSDNGAEDSTGPGPGNSRNSVRYGWKGERDTRGDSNWVPAE | 1.7398 |
| 4 | LATQSARDGPGPGLPIFDWGTRRANVKISETDQKIALSDGPGPGKTGDSPYEIGLDELSTGKGGPGPGNSRNSVRYGWKGERDTRGDSNWVPAEGPGPGSSTINEDPNSGTNNGNLTSGSCTPTTSDNGAEDST | 1.7499 |
| 5 | LATQSARDGPGPGLPIFDWGTRRANVKISETDQKIALSDGPGPGNSRNSVRYGWKGERDTRGDSNWVPAEGPGPGSSTINEDPNSGTNNGNLTSGSCTPTTSDNGAEDSTGPGPGKTGDSPYEIGLDELSTGKG | 1.6926 |
| 6 | LATQSARDGPGPGLPIFDWGTRRANVKISETDQKIALSDGPGPGNSRNSVRYGWKGERDTRGDSNWVPAEGPGPGKTGDSPYEIGLDELSTGKGGPGPGSSTINEDPNSGTNNGNLTSGSCTPTTSDNGAEDST | 1.7499 |
| 7 | LATQSARDGPGPGSSTINEDPNSGTNNGNLTSGSCTPTTSDNGAEDSTGPGPGLPIFDWGTRRANVKISETDQKIALSDGPGPGKTGDSPYEIGLDELSTGKGGPGPGNSRNSVRYGWKGERDTRGDSNWVPAE | 1.7398 |
| 8 | LATQSARDGPGPGSSTINEDPNSGTNNGNLTSGSCTPTTSDNGAEDSTGPGPGLPIFDWGTRRANVKISETDQKIALSDGPGPGNSRNSVRYGWKGERDTRGDSNWVPAEGPGPGKTGDSPYEIGLDELSTGKG | 1.6926 |
| 9 | LATQSARDGPGPGSSTINEDPNSGTNNGNLTSGSCTPTTSDNGAEDSTGPGPGKTGDSPYEIGLDELSTGKGGPGPGLPIFDWGTRRANVKISETDQKIALSDGPGPGNSRNSVRYGWKGERDTRGDSNWVPAE | 1.7398 |
| 10 | LATQSARDGPGPGSSTINEDPNSGTNNGNLTSGSCTPTTSDNGAEDSTGPGPGKTGDSPYEIGLDELSTGKGGPGPGNSRNSVRYGWKGERDTRGDSNWVPAEGPGPGLPIFDWGTRRANVKISETDQKIALSD | 1.7047 |
| 11 | LATQSARDGPGPGSSTINEDPNSGTNNGNLTSGSCTPTTSDNGAEDSTGPGPGNSRNSVRYGWKGERDTRGDSNWVPAEGPGPGLPIFDWGTRRANVKISETDQKIALSDGPGPGKTGDSPYEIGLDELSTGKG | 1.6926 |
| 12 | LATQSARDGPGPGSSTINEDPNSGTNNGNLTSGSCTPTTSDNGAEDSTGPGPGNSRNSVRYGWKGERDTRGDSNWVPAEGPGPGKTGDSPYEIGLDELSTGKGGPGPGLPIFDWGTRRANVKISETDQKIALSD | 1.7047 |
| 13 | LATQSARDGPGPGKTGDSPYEIGLDELSTGKGGPGPGLPIFDWGTRRANVKISETDQKIALSDGPGPGSSTINEDPNSGTNNGNLTSGSCTPTTSDNGAEDSTGPGPGNSRNSVRYGWKGERDTRGDSNWVPAE | 1.7398 |
| 14 | LATQSARDGPGPGKTGDSPYEIGLDELSTGKGGPGPGLPIFDWGTRRANVKISETDQKIALSDGPGPGNSRNSVRYGWKGERDTRGDSNWVPAEGPGPGSSTINEDPNSGTNNGNLTSGSCTPTTSDNGAEDST | 1.7499 |
| 15 | LATQSARDGPGPGKTGDSPYEIGLDELSTGKGGPGPGSSTINEDPNSGTNNGNLTSGSCTPTTSDNGAEDSTGPGPGLPIFDWGTRRANVKISETDQKIALSDGPGPGNSRNSVRYGWKGERDTRGDSNWVPAE | 1.7398 |
| 16 | LATQSARDGPGPGKTGDSPYEIGLDELSTGKGGPGPGSSTINEDPNSGTNNGNLTSGSCTPTTSDNGAEDSTGPGPGNSRNSVRYGWKGERDTRGDSNWVPAEGPGPGLPIFDWGTRRANVKISETDQKIALSD | 1.7047 |
| 17 | LATQSARDGPGPGKTGDSPYEIGLDELSTGKGGPGPGNSRNSVRYGWKGERDTRGDSNWVPAEGPGPGLPIFDWGTRRANVKISETDQKIALSDGPGPGSSTINEDPNSGTNNGNLTSGSCTPTTSDNGAEDST | 1.7499 |
| 18 | LATQSARDGPGPGKTGDSPYEIGLDELSTGKGGPGPGNSRNSVRYGWKGERDTRGDSNWVPAEGPGPGSSTINEDPNSGTNNGNLTSGSCTPTTSDNGAEDSTGPGPGLPIFDWGTRRANVKISETDQKIALSD | 1.7047 |
| 19 | LATQSARDGPGPGNSRNSVRYGWKGERDTRGDSNWVPAEGPGPGLPIFDWGTRRANVKISETDQKIALSDGPGPGSSTINEDPNSGTNNGNLTSGSCTPTTSDNGAEDSTGPGPGKTGDSPYEIGLDELSTGKG | 1.6926 |
| 20 | LATQSARDGPGPGNSRNSVRYGWKGERDTRGDSNWVPAEGPGPGLPIFDWGTRRANVKISETDQKIALSDGPGPGKTGDSPYEIGLDELSTGKGGPGPGSSTINEDPNSGTNNGNLTSGSCTPTTSDNGAEDST | 1.7499 |
| 21 | LATQSARDGPGPGNSRNSVRYGWKGERDTRGDSNWVPAEGPGPGSSTINEDPNSGTNNGNLTSGSCTPTTSDNGAEDSTGPGPGLPIFDWGTRRANVKISETDQKIALSDGPGPGKTGDSPYEIGLDELSTGKG | 1.6926 |
| 22 | LATQSARDGPGPGNSRNSVRYGWKGERDTRGDSNWVPAEGPGPGSSTINEDPNSGTNNGNLTSGSCTPTTSDNGAEDSTGPGPGKTGDSPYEIGLDELSTGKGGPGPGLPIFDWGTRRANVKISETDQKIALSD | 1.7047 |
| 23 | LATQSARDGPGPGNSRNSVRYGWKGERDTRGDSNWVPAEGPGPGKTGDSPYEIGLDELSTGKGGPGPGLPIFDWGTRRANVKISETDQKIALSDGPGPGSSTINEDPNSGTNNGNLTSGSCTPTTSDNGAEDST | 1.7499 |
| 24 | LATQSARDGPGPGNSRNSVRYGWKGERDTRGDSNWVPAEGPGPGKTGDSPYEIGLDELSTGKGGPGPGSSTINEDPNSGTNNGNLTSGSCTPTTSDNGAEDSTGPGPGLPIFDWGTRRANVKISETDQKIALSD | 1.7047 |
| 25 | LPIFDWGTRRANVKISETDQKIALSDGPGPGLATQSARDGPGPGSSTINEDPNSGTNNGNLTSGSCTPTTSDNGAEDSTGPGPGKTGDSPYEIGLDELSTGKGGPGPGNSRNSVRYGWKGERDTRGDSNWVPAE | 1.7433 |
| 26 | LPIFDWGTRRANVKISETDQKIALSDGPGPGLATQSARDGPGPGSSTINEDPNSGTNNGNLTSGSCTPTTSDNGAEDSTGPGPGNSRNSVRYGWKGERDTRGDSNWVPAEGPGPGKTGDSPYEIGLDELSTGKG | 1.6961 |
| 27 | LPIFDWGTRRANVKISETDQKIALSDGPGPGLATQSARDGPGPGKTGDSPYEIGLDELSTGKGGPGPGSSTINEDPNSGTNNGNLTSGSCTPTTSDNGAEDSTGPGPGNSRNSVRYGWKGERDTRGDSNWVPAE | 1.7433 |
| 28 | LPIFDWGTRRANVKISETDQKIALSDGPGPGLATQSARDGPGPGKTGDSPYEIGLDELSTGKGGPGPGNSRNSVRYGWKGERDTRGDSNWVPAEGPGPGSSTINEDPNSGTNNGNLTSGSCTPTTSDNGAEDST | 1.7534 |
| 29 | LPIFDWGTRRANVKISETDQKIALSDGPGPGLATQSARDGPGPGNSRNSVRYGWKGERDTRGDSNWVPAEGPGPGSSTINEDPNSGTNNGNLTSGSCTPTTSDNGAEDSTGPGPGKTGDSPYEIGLDELSTGKG | 1.6961 |
| 30 | LPIFDWGTRRANVKISETDQKIALSDGPGPGLATQSARDGPGPGNSRNSVRYGWKGERDTRGDSNWVPAEGPGPGKTGDSPYEIGLDELSTGKGGPGPGSSTINEDPNSGTNNGNLTSGSCTPTTSDNGAEDST | 1.7534 |
| 31 | LPIFDWGTRRANVKISETDQKIALSDGPGPGSSTINEDPNSGTNNGNLTSGSCTPTTSDNGAEDSTGPGPGLATQSARDGPGPGKTGDSPYEIGLDELSTGKGGPGPGNSRNSVRYGWKGERDTRGDSNWVPAE | 1.7433 |
| 32 | LPIFDWGTRRANVKISETDQKIALSDGPGPGSSTINEDPNSGTNNGNLTSGSCTPTTSDNGAEDSTGPGPGLATQSARDGPGPGNSRNSVRYGWKGERDTRGDSNWVPAEGPGPGKTGDSPYEIGLDELSTGKG | 1.6961 |
| 33 | LPIFDWGTRRANVKISETDQKIALSDGPGPGSSTINEDPNSGTNNGNLTSGSCTPTTSDNGAEDSTGPGPGKTGDSPYEIGLDELSTGKGGPGPGLATQSARDGPGPGNSRNSVRYGWKGERDTRGDSNWVPAE | 1.7433 |
| 34 | LPIFDWGTRRANVKISETDQKIALSDGPGPGSSTINEDPNSGTNNGNLTSGSCTPTTSDNGAEDSTGPGPGKTGDSPYEIGLDELSTGKGGPGPGNSRNSVRYGWKGERDTRGDSNWVPAEGPGPGLATQSARD | 1.7310 |
| 35 | LPIFDWGTRRANVKISETDQKIALSDGPGPGSSTINEDPNSGTNNGNLTSGSCTPTTSDNGAEDSTGPGPGNSRNSVRYGWKGERDTRGDSNWVPAEGPGPGLATQSARDGPGPGKTGDSPYEIGLDELSTGKG | 1.6961 |
| 36 | LPIFDWGTRRANVKISETDQKIALSDGPGPGSSTINEDPNSGTNNGNLTSGSCTPTTSDNGAEDSTGPGPGNSRNSVRYGWKGERDTRGDSNWVPAEGPGPGKTGDSPYEIGLDELSTGKGGPGPGLATQSARD | 1.7310 |
| 37 | LPIFDWGTRRANVKISETDQKIALSDGPGPGKTGDSPYEIGLDELSTGKGGPGPGLATQSARDGPGPGSSTINEDPNSGTNNGNLTSGSCTPTTSDNGAEDSTGPGPGNSRNSVRYGWKGERDTRGDSNWVPAE | 1.7433 |
| 38 | LPIFDWGTRRANVKISETDQKIALSDGPGPGKTGDSPYEIGLDELSTGKGGPGPGLATQSARDGPGPGNSRNSVRYGWKGERDTRGDSNWVPAEGPGPGSSTINEDPNSGTNNGNLTSGSCTPTTSDNGAEDST | 1.7534 |
| 39 | LPIFDWGTRRANVKISETDQKIALSDGPGPGKTGDSPYEIGLDELSTGKGGPGPGSSTINEDPNSGTNNGNLTSGSCTPTTSDNGAEDSTGPGPGLATQSARDGPGPGNSRNSVRYGWKGERDTRGDSNWVPAE | 1.7433 |
| 40 | LPIFDWGTRRANVKISETDQKIALSDGPGPGKTGDSPYEIGLDELSTGKGGPGPGSSTINEDPNSGTNNGNLTSGSCTPTTSDNGAEDSTGPGPGNSRNSVRYGWKGERDTRGDSNWVPAEGPGPGLATQSARD | 1.7310 |
| 41 | LPIFDWGTRRANVKISETDQKIALSDGPGPGKTGDSPYEIGLDELSTGKGGPGPGNSRNSVRYGWKGERDTRGDSNWVPAEGPGPGLATQSARDGPGPGSSTINEDPNSGTNNGNLTSGSCTPTTSDNGAEDST | 1.7534 |
| 42 | LPIFDWGTRRANVKISETDQKIALSDGPGPGKTGDSPYEIGLDELSTGKGGPGPGNSRNSVRYGWKGERDTRGDSNWVPAEGPGPGSSTINEDPNSGTNNGNLTSGSCTPTTSDNGAEDSTGPGPGLATQSARD | 1.7310 |
| 43 | LPIFDWGTRRANVKISETDQKIALSDGPGPGNSRNSVRYGWKGERDTRGDSNWVPAEGPGPGLATQSARDGPGPGSSTINEDPNSGTNNGNLTSGSCTPTTSDNGAEDSTGPGPGKTGDSPYEIGLDELSTGKG | 1.6961 |
| 44 | LPIFDWGTRRANVKISETDQKIALSDGPGPGNSRNSVRYGWKGERDTRGDSNWVPAEGPGPGLATQSARDGPGPGKTGDSPYEIGLDELSTGKGGPGPGSSTINEDPNSGTNNGNLTSGSCTPTTSDNGAEDST | 1.7534 |
| 45 | LPIFDWGTRRANVKISETDQKIALSDGPGPGNSRNSVRYGWKGERDTRGDSNWVPAEGPGPGSSTINEDPNSGTNNGNLTSGSCTPTTSDNGAEDSTGPGPGLATQSARDGPGPGKTGDSPYEIGLDELSTGKG | 1.6961 |
| 46 | LPIFDWGTRRANVKISETDQKIALSDGPGPGNSRNSVRYGWKGERDTRGDSNWVPAEGPGPGSSTINEDPNSGTNNGNLTSGSCTPTTSDNGAEDSTGPGPGKTGDSPYEIGLDELSTGKGGPGPGLATQSARD | 1.7310 |
| 47 | LPIFDWGTRRANVKISETDQKIALSDGPGPGNSRNSVRYGWKGERDTRGDSNWVPAEGPGPGKTGDSPYEIGLDELSTGKGGPGPGLATQSARDGPGPGSSTINEDPNSGTNNGNLTSGSCTPTTSDNGAEDST | 1.7534 |
| 48 | LPIFDWGTRRANVKISETDQKIALSDGPGPGNSRNSVRYGWKGERDTRGDSNWVPAEGPGPGKTGDSPYEIGLDELSTGKGGPGPGSSTINEDPNSGTNNGNLTSGSCTPTTSDNGAEDSTGPGPGLATQSARD | 1.7310 |
| 49 | SSTINEDPNSGTNNGNLTSGSCTPTTSDNGAEDSTGPGPGLATQSARDGPGPGLPIFDWGTRRANVKISETDQKIALSDGPGPGKTGDSPYEIGLDELSTGKGGPGPGNSRNSVRYGWKGERDTRGDSNWVPAE | 1.7264 |
| 50 | SSTINEDPNSGTNNGNLTSGSCTPTTSDNGAEDSTGPGPGLATQSARDGPGPGLPIFDWGTRRANVKISETDQKIALSDGPGPGNSRNSVRYGWKGERDTRGDSNWVPAEGPGPGKTGDSPYEIGLDELSTGKG | 1.6792 |
| 51 | SSTINEDPNSGTNNGNLTSGSCTPTTSDNGAEDSTGPGPGLATQSARDGPGPGKTGDSPYEIGLDELSTGKGGPGPGLPIFDWGTRRANVKISETDQKIALSDGPGPGNSRNSVRYGWKGERDTRGDSNWVPAE | 1.7264 |
| 52 | SSTINEDPNSGTNNGNLTSGSCTPTTSDNGAEDSTGPGPGLATQSARDGPGPGKTGDSPYEIGLDELSTGKGGPGPGNSRNSVRYGWKGERDTRGDSNWVPAEGPGPGLPIFDWGTRRANVKISETDQKIALSD | 1.6912 |
| 53 | SSTINEDPNSGTNNGNLTSGSCTPTTSDNGAEDSTGPGPGLATQSARDGPGPGNSRNSVRYGWKGERDTRGDSNWVPAEGPGPGLPIFDWGTRRANVKISETDQKIALSDGPGPGKTGDSPYEIGLDELSTGKG | 1.6792 |
| 54 | SSTINEDPNSGTNNGNLTSGSCTPTTSDNGAEDSTGPGPGLATQSARDGPGPGNSRNSVRYGWKGERDTRGDSNWVPAEGPGPGKTGDSPYEIGLDELSTGKGGPGPGLPIFDWGTRRANVKISETDQKIALSD | 1.6912 |
| 55 | SSTINEDPNSGTNNGNLTSGSCTPTTSDNGAEDSTGPGPGLPIFDWGTRRANVKISETDQKIALSDGPGPGLATQSARDGPGPGKTGDSPYEIGLDELSTGKGGPGPGNSRNSVRYGWKGERDTRGDSNWVPAE | 1.7264 |
| 56 | SSTINEDPNSGTNNGNLTSGSCTPTTSDNGAEDSTGPGPGLPIFDWGTRRANVKISETDQKIALSDGPGPGLATQSARDGPGPGNSRNSVRYGWKGERDTRGDSNWVPAEGPGPGKTGDSPYEIGLDELSTGKG | 1.6792 |
| 57 | SSTINEDPNSGTNNGNLTSGSCTPTTSDNGAEDSTGPGPGLPIFDWGTRRANVKISETDQKIALSDGPGPGKTGDSPYEIGLDELSTGKGGPGPGLATQSARDGPGPGNSRNSVRYGWKGERDTRGDSNWVPAE | 1.7264 |
| 58 | SSTINEDPNSGTNNGNLTSGSCTPTTSDNGAEDSTGPGPGLPIFDWGTRRANVKISETDQKIALSDGPGPGKTGDSPYEIGLDELSTGKGGPGPGNSRNSVRYGWKGERDTRGDSNWVPAEGPGPGLATQSARD | 1.7141 |
| 59 | SSTINEDPNSGTNNGNLTSGSCTPTTSDNGAEDSTGPGPGLPIFDWGTRRANVKISETDQKIALSDGPGPGNSRNSVRYGWKGERDTRGDSNWVPAEGPGPGLATQSARDGPGPGKTGDSPYEIGLDELSTGKG | 1.6792 |
| 60 | SSTINEDPNSGTNNGNLTSGSCTPTTSDNGAEDSTGPGPGLPIFDWGTRRANVKISETDQKIALSDGPGPGNSRNSVRYGWKGERDTRGDSNWVPAEGPGPGKTGDSPYEIGLDELSTGKGGPGPGLATQSARD | 1.7141 |
| 61 | SSTINEDPNSGTNNGNLTSGSCTPTTSDNGAEDSTGPGPGKTGDSPYEIGLDELSTGKGGPGPGLATQSARDGPGPGLPIFDWGTRRANVKISETDQKIALSDGPGPGNSRNSVRYGWKGERDTRGDSNWVPAE | 1.7264 |
| 62 | SSTINEDPNSGTNNGNLTSGSCTPTTSDNGAEDSTGPGPGKTGDSPYEIGLDELSTGKGGPGPGLATQSARDGPGPGNSRNSVRYGWKGERDTRGDSNWVPAEGPGPGLPIFDWGTRRANVKISETDQKIALSD | 1.6912 |
| 63 | SSTINEDPNSGTNNGNLTSGSCTPTTSDNGAEDSTGPGPGKTGDSPYEIGLDELSTGKGGPGPGLPIFDWGTRRANVKISETDQKIALSDGPGPGLATQSARDGPGPGNSRNSVRYGWKGERDTRGDSNWVPAE | 1.7264 |
| 64 | SSTINEDPNSGTNNGNLTSGSCTPTTSDNGAEDSTGPGPGKTGDSPYEIGLDELSTGKGGPGPGLPIFDWGTRRANVKISETDQKIALSDGPGPGNSRNSVRYGWKGERDTRGDSNWVPAEGPGPGLATQSARD | 1.7141 |
| 65 | SSTINEDPNSGTNNGNLTSGSCTPTTSDNGAEDSTGPGPGKTGDSPYEIGLDELSTGKGGPGPGNSRNSVRYGWKGERDTRGDSNWVPAEGPGPGLATQSARDGPGPGLPIFDWGTRRANVKISETDQKIALSD | 1.6912 |
| 66 | SSTINEDPNSGTNNGNLTSGSCTPTTSDNGAEDSTGPGPGKTGDSPYEIGLDELSTGKGGPGPGNSRNSVRYGWKGERDTRGDSNWVPAEGPGPGLPIFDWGTRRANVKISETDQKIALSDGPGPGLATQSARD | 1.7141 |
| 67 | SSTINEDPNSGTNNGNLTSGSCTPTTSDNGAEDSTGPGPGNSRNSVRYGWKGERDTRGDSNWVPAEGPGPGLATQSARDGPGPGLPIFDWGTRRANVKISETDQKIALSDGPGPGKTGDSPYEIGLDELSTGKG | 1.6792 |
| 68 | SSTINEDPNSGTNNGNLTSGSCTPTTSDNGAEDSTGPGPGNSRNSVRYGWKGERDTRGDSNWVPAEGPGPGLATQSARDGPGPGKTGDSPYEIGLDELSTGKGGPGPGLPIFDWGTRRANVKISETDQKIALSD | 1.6912 |
| 69 | SSTINEDPNSGTNNGNLTSGSCTPTTSDNGAEDSTGPGPGNSRNSVRYGWKGERDTRGDSNWVPAEGPGPGLPIFDWGTRRANVKISETDQKIALSDGPGPGLATQSARDGPGPGKTGDSPYEIGLDELSTGKG | 1.6792 |
| 70 | SSTINEDPNSGTNNGNLTSGSCTPTTSDNGAEDSTGPGPGNSRNSVRYGWKGERDTRGDSNWVPAEGPGPGLPIFDWGTRRANVKISETDQKIALSDGPGPGKTGDSPYEIGLDELSTGKGGPGPGLATQSARD | 1.7141 |
| 71 | SSTINEDPNSGTNNGNLTSGSCTPTTSDNGAEDSTGPGPGNSRNSVRYGWKGERDTRGDSNWVPAEGPGPGKTGDSPYEIGLDELSTGKGGPGPGLATQSARDGPGPGLPIFDWGTRRANVKISETDQKIALSD | 1.6912 |
| 72 | SSTINEDPNSGTNNGNLTSGSCTPTTSDNGAEDSTGPGPGNSRNSVRYGWKGERDTRGDSNWVPAEGPGPGKTGDSPYEIGLDELSTGKGGPGPGLPIFDWGTRRANVKISETDQKIALSDGPGPGLATQSARD | 1.7141 |
| 73 | KTGDSPYEIGLDELSTGKGGPGPGLATQSARDGPGPGLPIFDWGTRRANVKISETDQKIALSDGPGPGSSTINEDPNSGTNNGNLTSGSCTPTTSDNGAEDSTGPGPGNSRNSVRYGWKGERDTRGDSNWVPAE | 1.7671 |
| 74 | KTGDSPYEIGLDELSTGKGGPGPGLATQSARDGPGPGLPIFDWGTRRANVKISETDQKIALSDGPGPGNSRNSVRYGWKGERDTRGDSNWVPAEGPGPGSSTINEDPNSGTNNGNLTSGSCTPTTSDNGAEDST | 1.7772 |
| 75 | KTGDSPYEIGLDELSTGKGGPGPGLATQSARDGPGPGSSTINEDPNSGTNNGNLTSGSCTPTTSDNGAEDSTGPGPGLPIFDWGTRRANVKISETDQKIALSDGPGPGNSRNSVRYGWKGERDTRGDSNWVPAE | 1.7671 |
| 76 | KTGDSPYEIGLDELSTGKGGPGPGLATQSARDGPGPGSSTINEDPNSGTNNGNLTSGSCTPTTSDNGAEDSTGPGPGNSRNSVRYGWKGERDTRGDSNWVPAEGPGPGLPIFDWGTRRANVKISETDQKIALSD | 1.7319 |
| 77 | KTGDSPYEIGLDELSTGKGGPGPGLATQSARDGPGPGNSRNSVRYGWKGERDTRGDSNWVPAEGPGPGLPIFDWGTRRANVKISETDQKIALSDGPGPGSSTINEDPNSGTNNGNLTSGSCTPTTSDNGAEDST | 1.7772 |
| 78 | KTGDSPYEIGLDELSTGKGGPGPGLATQSARDGPGPGNSRNSVRYGWKGERDTRGDSNWVPAEGPGPGSSTINEDPNSGTNNGNLTSGSCTPTTSDNGAEDSTGPGPGLPIFDWGTRRANVKISETDQKIALSD | 1.7319 |
| 79 | KTGDSPYEIGLDELSTGKGGPGPGLPIFDWGTRRANVKISETDQKIALSDGPGPGLATQSARDGPGPGSSTINEDPNSGTNNGNLTSGSCTPTTSDNGAEDSTGPGPGNSRNSVRYGWKGERDTRGDSNWVPAE | 1.7671 |
| 80 | KTGDSPYEIGLDELSTGKGGPGPGLPIFDWGTRRANVKISETDQKIALSDGPGPGLATQSARDGPGPGNSRNSVRYGWKGERDTRGDSNWVPAEGPGPGSSTINEDPNSGTNNGNLTSGSCTPTTSDNGAEDST | 1.7772 |
| 81 | KTGDSPYEIGLDELSTGKGGPGPGLPIFDWGTRRANVKISETDQKIALSDGPGPGSSTINEDPNSGTNNGNLTSGSCTPTTSDNGAEDSTGPGPGLATQSARDGPGPGNSRNSVRYGWKGERDTRGDSNWVPAE | 1.7671 |
| 82 | KTGDSPYEIGLDELSTGKGGPGPGLPIFDWGTRRANVKISETDQKIALSDGPGPGSSTINEDPNSGTNNGNLTSGSCTPTTSDNGAEDSTGPGPGNSRNSVRYGWKGERDTRGDSNWVPAEGPGPGLATQSARD | 1.7548 |
| 83 | KTGDSPYEIGLDELSTGKGGPGPGLPIFDWGTRRANVKISETDQKIALSDGPGPGNSRNSVRYGWKGERDTRGDSNWVPAEGPGPGLATQSARDGPGPGSSTINEDPNSGTNNGNLTSGSCTPTTSDNGAEDST | 1.7772 |
| 84 | KTGDSPYEIGLDELSTGKGGPGPGLPIFDWGTRRANVKISETDQKIALSDGPGPGNSRNSVRYGWKGERDTRGDSNWVPAEGPGPGSSTINEDPNSGTNNGNLTSGSCTPTTSDNGAEDSTGPGPGLATQSARD | 1.7548 |
| 85 | KTGDSPYEIGLDELSTGKGGPGPGSSTINEDPNSGTNNGNLTSGSCTPTTSDNGAEDSTGPGPGLATQSARDGPGPGLPIFDWGTRRANVKISETDQKIALSDGPGPGNSRNSVRYGWKGERDTRGDSNWVPAE | 1.7671 |
| 86 | KTGDSPYEIGLDELSTGKGGPGPGSSTINEDPNSGTNNGNLTSGSCTPTTSDNGAEDSTGPGPGLATQSARDGPGPGNSRNSVRYGWKGERDTRGDSNWVPAEGPGPGLPIFDWGTRRANVKISETDQKIALSD | 1.7319 |
| 87 | KTGDSPYEIGLDELSTGKGGPGPGSSTINEDPNSGTNNGNLTSGSCTPTTSDNGAEDSTGPGPGLPIFDWGTRRANVKISETDQKIALSDGPGPGLATQSARDGPGPGNSRNSVRYGWKGERDTRGDSNWVPAE | 1.7671 |
| 88 | KTGDSPYEIGLDELSTGKGGPGPGSSTINEDPNSGTNNGNLTSGSCTPTTSDNGAEDSTGPGPGLPIFDWGTRRANVKISETDQKIALSDGPGPGNSRNSVRYGWKGERDTRGDSNWVPAEGPGPGLATQSARD | 1.7548 |
| 89 | KTGDSPYEIGLDELSTGKGGPGPGSSTINEDPNSGTNNGNLTSGSCTPTTSDNGAEDSTGPGPGNSRNSVRYGWKGERDTRGDSNWVPAEGPGPGLATQSARDGPGPGLPIFDWGTRRANVKISETDQKIALSD | 1.7319 |
| 90 | KTGDSPYEIGLDELSTGKGGPGPGSSTINEDPNSGTNNGNLTSGSCTPTTSDNGAEDSTGPGPGNSRNSVRYGWKGERDTRGDSNWVPAEGPGPGLPIFDWGTRRANVKISETDQKIALSDGPGPGLATQSARD | 1.7548 |
| 91 | KTGDSPYEIGLDELSTGKGGPGPGNSRNSVRYGWKGERDTRGDSNWVPAEGPGPGLATQSARDGPGPGLPIFDWGTRRANVKISETDQKIALSDGPGPGSSTINEDPNSGTNNGNLTSGSCTPTTSDNGAEDST | 1.7772 |
| 92 | KTGDSPYEIGLDELSTGKGGPGPGNSRNSVRYGWKGERDTRGDSNWVPAEGPGPGLATQSARDGPGPGSSTINEDPNSGTNNGNLTSGSCTPTTSDNGAEDSTGPGPGLPIFDWGTRRANVKISETDQKIALSD | 1.7319 |
| 93 | KTGDSPYEIGLDELSTGKGGPGPGNSRNSVRYGWKGERDTRGDSNWVPAEGPGPGLPIFDWGTRRANVKISETDQKIALSDGPGPGLATQSARDGPGPGSSTINEDPNSGTNNGNLTSGSCTPTTSDNGAEDST | 1.7772 |
| 94 | KTGDSPYEIGLDELSTGKGGPGPGNSRNSVRYGWKGERDTRGDSNWVPAEGPGPGLPIFDWGTRRANVKISETDQKIALSDGPGPGSSTINEDPNSGTNNGNLTSGSCTPTTSDNGAEDSTGPGPGLATQSARD | 1.7548 |
| 95 | KTGDSPYEIGLDELSTGKGGPGPGNSRNSVRYGWKGERDTRGDSNWVPAEGPGPGSSTINEDPNSGTNNGNLTSGSCTPTTSDNGAEDSTGPGPGLATQSARDGPGPGLPIFDWGTRRANVKISETDQKIALSD | 1.7319 |
| 96 | KTGDSPYEIGLDELSTGKGGPGPGNSRNSVRYGWKGERDTRGDSNWVPAEGPGPGSSTINEDPNSGTNNGNLTSGSCTPTTSDNGAEDSTGPGPGLPIFDWGTRRANVKISETDQKIALSDGPGPGLATQSARD | 1.7548 |
| 97 | NSRNSVRYGWKGERDTRGDSNWVPAEGPGPGLATQSARDGPGPGLPIFDWGTRRANVKISETDQKIALSDGPGPGSSTINEDPNSGTNNGNLTSGSCTPTTSDNGAEDSTGPGPGKTGDSPYEIGLDELSTGKG | 1.8239 |
| 98 | NSRNSVRYGWKGERDTRGDSNWVPAEGPGPGLATQSARDGPGPGLPIFDWGTRRANVKISETDQKIALSDGPGPGKTGDSPYEIGLDELSTGKGGPGPGSSTINEDPNSGTNNGNLTSGSCTPTTSDNGAEDST | 1.8811 |
| 99 | NSRNSVRYGWKGERDTRGDSNWVPAEGPGPGLATQSARDGPGPGSSTINEDPNSGTNNGNLTSGSCTPTTSDNGAEDSTGPGPGLPIFDWGTRRANVKISETDQKIALSDGPGPGKTGDSPYEIGLDELSTGKG | 1.8239 |
| 100 | NSRNSVRYGWKGERDTRGDSNWVPAEGPGPGLATQSARDGPGPGSSTINEDPNSGTNNGNLTSGSCTPTTSDNGAEDSTGPGPGKTGDSPYEIGLDELSTGKGGPGPGLPIFDWGTRRANVKISETDQKIALSD | 1.8359 |
| 101 | NSRNSVRYGWKGERDTRGDSNWVPAEGPGPGLATQSARDGPGPGKTGDSPYEIGLDELSTGKGGPGPGLPIFDWGTRRANVKISETDQKIALSDGPGPGSSTINEDPNSGTNNGNLTSGSCTPTTSDNGAEDST | 1.8811 |
| 102 | NSRNSVRYGWKGERDTRGDSNWVPAEGPGPGLATQSARDGPGPGKTGDSPYEIGLDELSTGKGGPGPGSSTINEDPNSGTNNGNLTSGSCTPTTSDNGAEDSTGPGPGLPIFDWGTRRANVKISETDQKIALSD | 1.8359 |
| 103 | NSRNSVRYGWKGERDTRGDSNWVPAEGPGPGLPIFDWGTRRANVKISETDQKIALSDGPGPGLATQSARDGPGPGSSTINEDPNSGTNNGNLTSGSCTPTTSDNGAEDSTGPGPGKTGDSPYEIGLDELSTGKG | 1.8239 |
| 104 | NSRNSVRYGWKGERDTRGDSNWVPAEGPGPGLPIFDWGTRRANVKISETDQKIALSDGPGPGLATQSARDGPGPGKTGDSPYEIGLDELSTGKGGPGPGSSTINEDPNSGTNNGNLTSGSCTPTTSDNGAEDST | 1.8811 |
| 105 | NSRNSVRYGWKGERDTRGDSNWVPAEGPGPGLPIFDWGTRRANVKISETDQKIALSDGPGPGSSTINEDPNSGTNNGNLTSGSCTPTTSDNGAEDSTGPGPGLATQSARDGPGPGKTGDSPYEIGLDELSTGKG | 1.8239 |
| 106 | NSRNSVRYGWKGERDTRGDSNWVPAEGPGPGLPIFDWGTRRANVKISETDQKIALSDGPGPGSSTINEDPNSGTNNGNLTSGSCTPTTSDNGAEDSTGPGPGKTGDSPYEIGLDELSTGKGGPGPGLATQSARD | 1.8588 |
| 107 | NSRNSVRYGWKGERDTRGDSNWVPAEGPGPGLPIFDWGTRRANVKISETDQKIALSDGPGPGKTGDSPYEIGLDELSTGKGGPGPGLATQSARDGPGPGSSTINEDPNSGTNNGNLTSGSCTPTTSDNGAEDST | 1.8811 |
| 108 | NSRNSVRYGWKGERDTRGDSNWVPAEGPGPGLPIFDWGTRRANVKISETDQKIALSDGPGPGKTGDSPYEIGLDELSTGKGGPGPGSSTINEDPNSGTNNGNLTSGSCTPTTSDNGAEDSTGPGPGLATQSARD | 1.8588 |
| 109 | NSRNSVRYGWKGERDTRGDSNWVPAEGPGPGSSTINEDPNSGTNNGNLTSGSCTPTTSDNGAEDSTGPGPGLATQSARDGPGPGLPIFDWGTRRANVKISETDQKIALSDGPGPGKTGDSPYEIGLDELSTGKG | 1.8239 |
| 110 | NSRNSVRYGWKGERDTRGDSNWVPAEGPGPGSSTINEDPNSGTNNGNLTSGSCTPTTSDNGAEDSTGPGPGLATQSARDGPGPGKTGDSPYEIGLDELSTGKGGPGPGLPIFDWGTRRANVKISETDQKIALSD | 1.8359 |
| 111 | NSRNSVRYGWKGERDTRGDSNWVPAEGPGPGSSTINEDPNSGTNNGNLTSGSCTPTTSDNGAEDSTGPGPGLPIFDWGTRRANVKISETDQKIALSDGPGPGLATQSARDGPGPGKTGDSPYEIGLDELSTGKG | 1.8239 |
| 112 | NSRNSVRYGWKGERDTRGDSNWVPAEGPGPGSSTINEDPNSGTNNGNLTSGSCTPTTSDNGAEDSTGPGPGLPIFDWGTRRANVKISETDQKIALSDGPGPGKTGDSPYEIGLDELSTGKGGPGPGLATQSARD | 1.8588 |
| 113 | NSRNSVRYGWKGERDTRGDSNWVPAEGPGPGSSTINEDPNSGTNNGNLTSGSCTPTTSDNGAEDSTGPGPGKTGDSPYEIGLDELSTGKGGPGPGLATQSARDGPGPGLPIFDWGTRRANVKISETDQKIALSD | 1.8359 |
| 114 | NSRNSVRYGWKGERDTRGDSNWVPAEGPGPGSSTINEDPNSGTNNGNLTSGSCTPTTSDNGAEDSTGPGPGKTGDSPYEIGLDELSTGKGGPGPGLPIFDWGTRRANVKISETDQKIALSDGPGPGLATQSARD | 1.8588 |
| 115 | NSRNSVRYGWKGERDTRGDSNWVPAEGPGPGKTGDSPYEIGLDELSTGKGGPGPGLATQSARDGPGPGLPIFDWGTRRANVKISETDQKIALSDGPGPGSSTINEDPNSGTNNGNLTSGSCTPTTSDNGAEDST | 1.8811 |
| 116 | NSRNSVRYGWKGERDTRGDSNWVPAEGPGPGKTGDSPYEIGLDELSTGKGGPGPGLATQSARDGPGPGSSTINEDPNSGTNNGNLTSGSCTPTTSDNGAEDSTGPGPGLPIFDWGTRRANVKISETDQKIALSD | 1.8359 |
| 117 | NSRNSVRYGWKGERDTRGDSNWVPAEGPGPGKTGDSPYEIGLDELSTGKGGPGPGLPIFDWGTRRANVKISETDQKIALSDGPGPGLATQSARDGPGPGSSTINEDPNSGTNNGNLTSGSCTPTTSDNGAEDST | 1.8811 |
| 118 | NSRNSVRYGWKGERDTRGDSNWVPAEGPGPGKTGDSPYEIGLDELSTGKGGPGPGLPIFDWGTRRANVKISETDQKIALSDGPGPGSSTINEDPNSGTNNGNLTSGSCTPTTSDNGAEDSTGPGPGLATQSARD | 1.8588 |
| 119 | NSRNSVRYGWKGERDTRGDSNWVPAEGPGPGKTGDSPYEIGLDELSTGKGGPGPGSSTINEDPNSGTNNGNLTSGSCTPTTSDNGAEDSTGPGPGLATQSARDGPGPGLPIFDWGTRRANVKISETDQKIALSD | 1.8359 |
| 120 | NSRNSVRYGWKGERDTRGDSNWVPAEGPGPGKTGDSPYEIGLDELSTGKGGPGPGSSTINEDPNSGTNNGNLTSGSCTPTTSDNGAEDSTGPGPGLPIFDWGTRRANVKISETDQKIALSDGPGPGLATQSARD | 1.8588 |


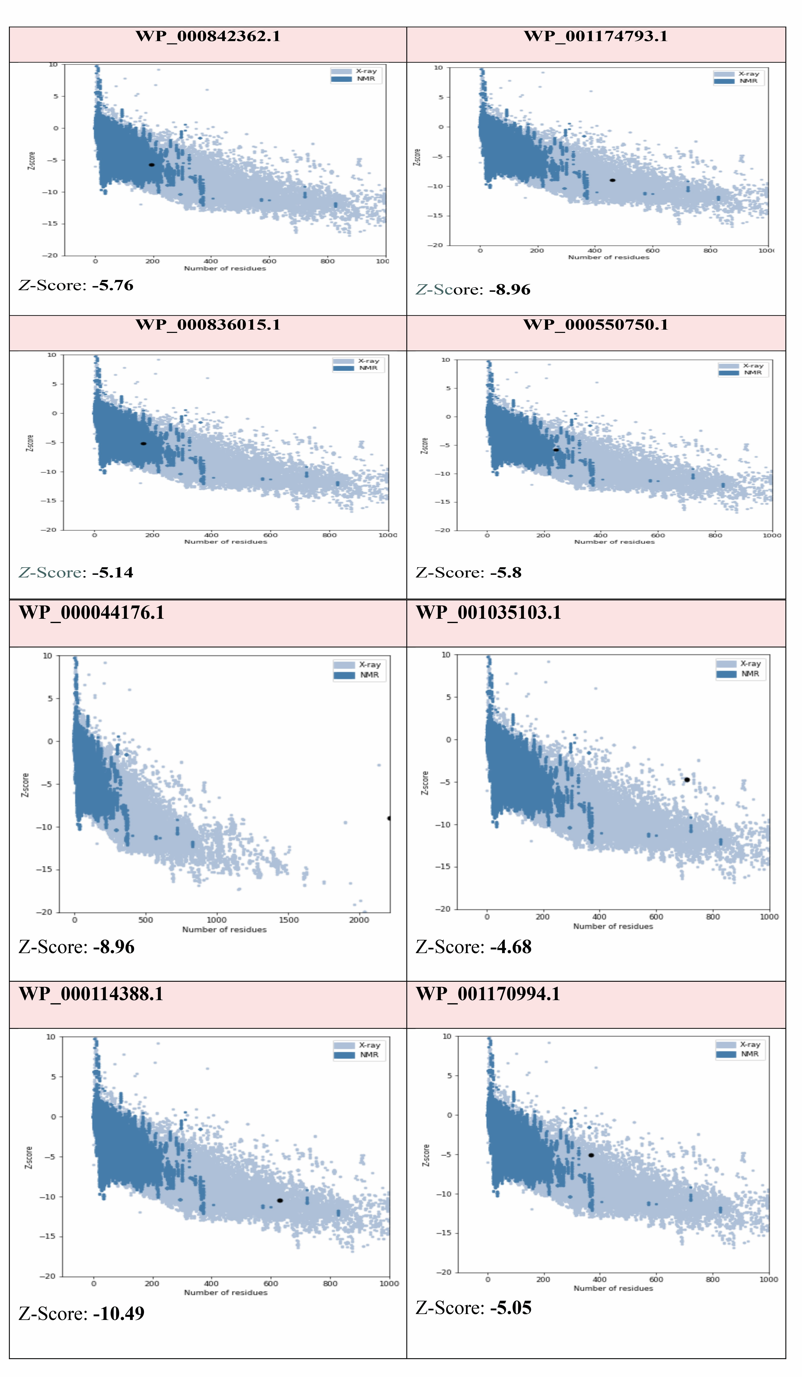


**Supplementary Figure 1:** ProSA-web analysis of eight multi-epitope constructs.
