# Supplementary material for: Development of a multi-epitope vaccine against Acinetobacter baumannii: A comprehensive approach to combating antimicrobial resistance
Source: PLoS One. 2025 Mar 10;20(3):e0319191. doi: 10.1371/journal.pone.0319191 (PMC11892874; doi:10.1371/journal.pone.0319191)
Supplement: Attachment — Submitted filename: Response to reviewers.docx [file pone.0319191.s004.docx]

**Dear Reviewer 1**

First, we would like to thank you for your very careful review of our paper and for your valuable comments, corrections, and suggestions as well. All the revised sections in the manuscript have been highlighted in yellow for your convenience. We hope that these changes enhance the clarity and comprehensibility of our study and address your concerns effectively.

Once again, we sincerely thank you for your diligent review, and we are committed to ensuring that our manuscript meets the highest standards of quality and accuracy.

Here below my corrections/suggestions:

Reviewer #1: The manuscript presents a comprehensive study focused on the development of a ligand-based drug design approach targeting infectious diseases. The authors have employed molecular docking and molecular dynamics simulations to predict the efficacy of potential drug candidates. The research is of high relevance and could contribute significantly to the field of drug discovery. However, several areas in the manuscript require further clarification and enhancement to meet the standards of publication.

1. The introduction provides a good background on the topic, but it could benefit from additional references to recent studies that have successfully applied ligand-based drug design in the context of infectious diseases. Including examples from these studies would add depth and context to the manuscript. Referencing recent studies for examples of ligand-based drug design in infectious disease research would add depth (<https://doi.org/10.1371/journal.pone.0302390>).

Response: We have revised the introduction by including recent studies demonstrating the successful use of LBDD in various infectious disease contexts. Specifically, we added a passage in lines **77-86**, highlighting the application of LBDD in identifying novel inhibitors for *Schistosoma mansoni* (Ja’afaru et al., 2024), tuberculosis, malaria, and viral infections like Zika (Capela et al., 2023; Kumar et al., 2022; Ramamurthy et al., 2021; Rao et al., 2023; Wong et al., 2023).

Furthermore, we extended this context to our study, which applies LBDD to *Acinetobacter baumannii*, a multidrug-resistant superbug. Our research uses advanced computational tools and bioinformatics to discover novel vaccine candidates, identify druggable targets, and propose new strategies for combating *A. baumannii*-related infections. This addition provides a more comprehensive background and enhances the depth of the manuscript as suggested.

2. Include molecular dynamics simulation studies to validate the docking results. Use Gromacs or Yasara to run the 100/200 ns simulation. The parameters used for docking, such as grid size and docking scores, should be explicitly stated. The molecular dynamics simulation parameters, including the force field, simulation time, and system setup, should be detailed. The authors should also discuss the stability criteria for the protein-ligand complexes. Including references to recent studies on similar methodologies would enhance this section (<https://doi.org/10.1371/journal.pone.0302440>, <https://doi.org/10.1038/s41598-024-61074-7>, <https://doi.org/10.1371/journal.pone.0300778>),
 (<https://doi.org/10.1080/07391102.2020.1856944>, <https://doi.org/10.5808/gi.20068>).


Response: We have implemented the recommended molecular dynamics (MD) simulation studies to validate the docking results. Specifically, we conducted 100 ns MD simulations using Gromacs 2019, and the detailed methodology has been incorporated into the **Methods** section. We also provided comprehensive details on the MD simulation setup, including the force field, simulation time, and system configuration in the **Abstract (Lines 46-49)**, and **Methods** (**lines 257-275**), with corresponding updates in the **Results** (**lines 400-414**), **Discussion (Lines 512-541)**, and **Conclusion** (**lines 628-629**). In addition, we have explicitly included the docking parameters, such as grid size and docking scores, in the **Methods** (**lines 218-228**) and **Results** (**lines 390-399**).

Furthermore, the stability criteria for protein-ligand complexes were discussed in the **Discussion** section (**lines 523-541**). To enhance the rigor of our work, we referenced recent studies on similar methodologies (<https://doi.org/10.1371/journal.pone.0302440>, <https://doi.org/10.1038/s41598-024-61074-7>, <https://doi.org/10.1371/journal.pone.0300778>, <https://doi.org/10.5808/gi.20068>).

We also included Figure 6, which presents the MD simulation results and highlights the RMSD, RMSF, and RG analyses.

3. While the study demonstrates promising computational predictions, validating these predictions through in vitro or in vivo experim. The authors should discuss plans for future experimental validation and consider citing comprehensive validation processes in similar research (<https://doi.org/10.1371/journal.pone.0301519>, <https://doi.org/10.1080/07391102.2022.2037041>).

Response: In response to your suggestion, we have added a comprehensive section in the discussion (**lines 580-604**) detailing our plans for future experimental validation, including both *in vitro* and *in vivo* approaches.

Additionally, we have incorporated references to similar research that emphasize thorough validation processes, including the studies mentioned in your comment (https://doi.org/10.1371/journal.pone.0301519, https://doi.org/10.1093/nar/gkab279, https://doi.org/10.3389/fimmu.2023.1259612, https://doi.org/10.1371/journal.pone.0196484, https://doi.org/10.1371/journal.pone.0294663, https://doi.org/10.1021/acsomega.9b00944).

4. The graphical abstract and figures could be more informative by including more labels and detailed legends. This would help in understanding the complex processes and results. Referencing a well-designed graphical abstract may provide a useful template (<https://doi.org/10.1016/j.heliyon.2023.e08002>).

Response: We have revised all figures to include more informative labels and detailed legends.

5. The results section should provide clearer explanations of the docking scores and molecular dynamics results. Discussing the implications of the results in a broader context would help readers understand their significance. Providing additional context and comparison for the results could be beneficial (<https://doi.org/10.1038/s41598-022-07553-7>, <https://doi.org/10.1016/j.imu.2021.101069>).

Response: We have provided more detailed explanations of the docking scores. The revisions are in **lines 390-399**, where we now clearly describe how the docking scores correlate with binding affinities and stability predictions. Additionally, we have expanded the result explanation of the MD simulations. These updates are located in lines **400-414**.

Although we could not access the specific references you provided, we have included detailed descriptions of the MD methodology used in our study. These explanations appear in the Methods section (**lines 257-275**) and provide a thorough overview of the techniques employed, ensuring transparency and replicability for future research.

We have also expanded the discussion of the results to include a broader context, helping readers understand their significance in the field (**lines 512-541).**

6. A more detailed description of the computational tools and software versions used in the study would enhance reproducibility. Including information on tool settings and configurations is essential. For examples of detailed computational methodologies, see recent studies (https://doi.org/10.3389/fimmu.2022.863234, https://doi.org/10.1016/j.imu.2021.100500).

Response: In response to your suggestion that we provide a more detailed description of the computational tools and software versions used, we have revised the Methods section to provide a more comprehensive account, including specific software versions, settings, and configurations applied during the analyses.

We have included the exact versions of all computational tools used in the study, such as AlgPred v2.0, AllerTOP v2.0, VaxiJen v2.0, Protein-Sol v2.0, ExPASy ProtParam, etc.

We have provided detailed information on the settings and configurations applied in each tool. For example, we specified using a cutoff value of 0.5 for allergenicity predictions in AlgPred, a threshold of 0.4 in VaxiJen for antigenicity, and a solubility score of 0.45 in Protein-Sol. In instances where default settings were used, we made sure to state that as well explicitly.

These revisions align with the examples of detailed computational methodologies provided in recent studies (https://doi.org/10.3389/fimmu.2022.863234, https://doi.org/10.1016/j.imu.2021.100500). All of these sections have been highlighted in the main body of the text.

7. The discussion should include a more comprehensive analysis of the study's limitations and the potential impact on the findings. Discussing the limitations openly will provide a balanced view of the study's contributions and areas for improvement.

Response: We have added a section addressing the limitations of the study in **lines 605-619**.

**Dear Reviewer 2**

First, we would like to thank you for your very careful review of our paper and for your subsequent comments, corrections, and suggestions as well. All the revised parts in the manuscript file are highlighted in red.

Reviewer #2: Manuscript entitled “Uncovering Novel Vaccine Candidates and Drug Targets in Acinetobacter baumannii: A New Approach to Fighting against the Superbug” developed a multi epitope vaccine and identified novel drug targets for A. baumannii, providing broad protection against MDR A. baumannii strains. The MEV showed strong potential through molecular docking. Though the study is good and will contribute to scientific society still there are some points, which need to be addressed before considering it for publication.


1. “A new approach” in title does not justify the approaches used by the authors. As both approaches used to identify drug and vaccine targets, have already used by many researchers. Thus, I suggest modifying the title a bit.

Response: We have revised the title to reflect better the nature of the approaches used and avoid the implication of novelty in the methodology. The new title, " **Development of a Multi-Epitope Vaccine and Identification of Novel Drug Targets Against *Acinetobacter baumannii*: A Comprehensive Approach to Combating Antimicrobial Resistance**," accurately represents the study's focus without suggesting that the approaches are entirely new. This change aligns with the reviewer's suggestion.

2. Choice of strain should be explained. If it is a reference sequence, please mention RefSeq ID or else the interest to choose the particular strain should be clearly mentioned.

Response: We have revised the Methods section to provide a more detailed explanation for our strain choice. Based on an analysis of published articles, we selected Acinetobacter baumannii VB7036 due to its clinically relevant characteristics. This strain belongs to the globally widespread, multidrug-resistant Sequence Type 2 (ST2) lineage, frequently associated with healthcare-associated infections. Additionally, it was isolated from a patient with bacteremia in India in 2019, adding further significance for studying antibiotic resistance mechanisms. We have included the GenBank accession number CP050523 to ensure transparency and reproducibility. Please refer to **lines 107-115** for the updated details.

3. Line 153, “Proteins that were more than the 75% standard were selected for further analysis.” Is not clear to understand.

Response: Thank you for your comment. We have revised the sentence in section 2.1.5. in **lines 138-144** to improve clarity.

4. Results of section 2.1.5, i.e., finding conserved proteins among all available strains, have not been found in the results section.

Response: Thank you for your comment. To address this, we have added a new section, 3.1.3. Prevalence of Putative Immunogenic Targets Among Circulating *A. baumannii* Strains, in **lines** **335-337**. This section provides a detailed explanation of the selection criteria for conserved proteins.

5. In heading “3.2.3. Choosing Critical Protein Sequences” It should be essential proteins instead of critical.

Response: We have changed the heading “3.2.3. Choosing Critical Protein Sequences” to “3.2.3. Choosing Essential Protein Sequences (**line 426**).

6. Line 376 “A. baumannii” should be italicized.

Response: We have reviewed the entire manuscript and corrected the formatting of *A. baumannii* throughout the text.

7. Targets identified as “potential drug targets” should be discussed in details as how and why these should be targeted. How their respective pathways can lead to recovery and how drugs can stimulate their pathway to support your results.

Response: In response to your comment, we have added a detailed discussion of the identified potential drug targets in **lines 551-579** of the manuscript. This new section explains in depth how each of the five targets identified in our study contributes to the biology and survival of *A. baumannii* and why they are promising targets for therapeutic intervention.

The proteins identified, including the glycosyl hydrolase 108 family protein (WP_001019691.1), anti-sigma factor GigB (WP_000103064.1), DUF5713 family protein (WP_000461267.1), and two hypothetical proteins (WP_000269705.1 and WP_000580181.1), were selected based on their essential roles in the bacterium’s survival mechanisms and their non-homology to human proteins, reducing the risk of off-target effects in humans.

Each target has been carefully evaluated for its role in key pathways such as carbohydrate metabolism, transcriptional regulation, and oxidative stress response. We explained how inhibiting these proteins could disrupt vital processes like cell wall biosynthesis, energy production, and gene expression in *A. baumannii*, leading to bacterial death or weakened resistance. We also discuss the potential of drugs to interact with these proteins, either through enzyme inhibition or interference with protein-protein interactions, to support bacterial clearance and aid in patient recovery.

8. As authors have also mentioned that many studies have already predicted MEVs against the said pathogen, then there should be a very clear description on the importance of current study and how it stands out already proposed vaccine candidates prioritized using same approach.

Response: In response to your comment, we have added a section in **lines 485-511** of the discussion that clarifies how our study distinguishes itself from similar multi-epitope vaccine (MEV) design studies, such as <https://doi.org/10.1186/s12864-024-10691-7>, [10.1007/s10989-021-10316-7](https://doi.org/10.1007%2Fs10989-021-10316-7), <https://doi.org/10.1007/s10989-021-10316-7>.

While those studies used in silico methods for *A. baumannii* vaccine design, our approach focuses on conserved outer membrane proteins (OMPs), explicitly targeting extracellular loop regions. We identified surface-exposed and conserved B-cell epitopes and then enhanced their immunogenicity through epitope shuffling.

Furthermore, the selected epitopes, derived from four conserved proteins, demonstrated over 75% prevalence and 100% sequence conservation across diverse *A. baumannii* strains, ensuring robust and wide-ranging protection. This high level of conservation optimizes immunogenicity and broadens the vaccine’s protective scope compared to previous works. These innovative aspects, coupled with the inclusion of detailed protein-protein interaction analyses and TLR binding studies, ensure the elicitation of both humoral and cellular immune responses.
