# Supplementary material for: Development of a multi-epitope vaccine against Acinetobacter baumannii: A comprehensive approach to combating antimicrobial resistance
Source: PLoS One. 2025 Mar 10;20(3):e0319191. doi: 10.1371/journal.pone.0319191 (PMC11892874; doi:10.1371/journal.pone.0319191)
Supplement: Attachment — Submitted filename: Response.docx [file pone.0319191.s005.docx]

**Dear reviewer,**

**Thank you for your constructive comments; all your comments applied to the manuscript have been highlighted in yellow.**

Reviewer #1: Thank you for submitting your manuscript, which provides interesting insights into your research. While the overall framework of the study is solid, I find that the molecular dynamics simulation (MDS) section requires further development to enhance the study's depth and scientific rigor.

In particular, the simulation could be improved by extending the duration to at least 100 nanoseconds, using tools such as GROMACS or Yasara. Additionally, key analyses like Root Mean Square Deviation (RMSD), Root Mean Square Fluctuation (RMSF), Radius of Gyration (Rg), hydrogen bond analysis (H-BOND), and Principal Component Analysis (PCA) are notably absent and should be included. These analyses are crucial for a more thorough interpretation of the molecular dynamics results.

All the changes made in response to the reviewer’s comment, including the extended simulation, additional analyses, and revised figures and manuscript sections, are highlighted in yellow in the updated manuscript.

Response: We thank the reviewer for their constructive feedback. In response, we have extended the molecular dynamics simulation to 100 nanoseconds (100,000 ps) using GROMACS, as suggested, and have incorporated key analyses such as Root Mean Square Deviation (RMSD), Root Mean Square Fluctuation (RMSF), Radius of Gyration (Rg), Hydrogen Bond Analysis (H-Bond), and Principal Component Analysis (PCA) to enhance the depth of our study. Specifically, we performed hydrogen bond analysis and PCA. The results, along with updated figures (Fig. 6D and Fig. 7), and the revised manuscript sections (lines 275–289 in Methods, 441–457 in Results, and lines 419–425) now provide a more thorough interpretation of the molecular dynamics simulations.

**Thank you for your constructive comment, all relevant sentence in manuscript was revised which have been highlighted in green.**

**1****.** Reviewer #3: “Eight OMPs isolated from A. baumanniiA. baumannii, were identified as potential 44 immunogenic targets. MEVs were designed using five critical epitopes from four proteins, 45 highlighting their potential as vaccine candidates.” Why so? Out eight why five were used for the vaccine design.

Response: To address the reviewer's query regarding the selection of five epitopes from four proteins out of the initial eight identified, we employed a rigorous, multi-step selection process to ensure the inclusion of the most promising candidates in our multi-epitope vaccine (MEV) construct. Initially, we analyzed the proteome of *Acinetobacter baumannii* strain VB7036, comprising 3,752 proteins, to identify 138 surface-exposed proteins, including extracellular and outer membrane proteins (OMPs), which are more likely to interact with the immune system. Among these, 68 proteins exhibited antigenicity scores of ≥0.5, indicating their potential to elicit strong immune responses. To minimize the risk of allergic reactions, we utilized the AlgPred tool, resulting in 57 non-allergenic proteins. Subsequently, we excluded 10 proteins with >30% sequence identity to human proteins to prevent autoimmune responses, leaving 47 non-homologous proteins. We then assessed the prevalence of these proteins across 560 circulating *A. baumannii* strains, excluding three proteins present in less than 75% of strains, thus narrowing the pool to 44 proteins. Further evaluation involved calculating the ratio of linear B-cell epitopes to MHC-II binding sites, identifying 13 proteins with favorable ratios (<0.5) and molecular weights under 110 kDa, suggesting their suitability to induce both humoral and cellular immune responses. Physicochemical analyses led to the exclusion of five proteins with unfavorable characteristics, resulting in eight proteins deemed optimal for vaccine development. Ultimately, we selected five B-cell epitopes from four proteins for the MEV construct based on stringent criteria, including high antigenicity (VaxiJen scores ≥0.5), non-allergenicity (confirmed by AllerTOP and AlgPred), 100% sequence conservancy across multiple *A. baumannii* strains, optimal epitope lengths (8–20 amino acids), strong binding affinity to MHC molecules (IC50 values <100 nM), appropriate molecular weight, minimal toxicity, no cross-reactivity with human proteins, favorable physicochemical properties (solubility score: 0.844), minimal transmembrane regions, structural integrity, and ease of synthesis and expression. This meticulous selection process ensured that the final MEV construct comprises epitopes with the highest potential to induce a robust and safe immune response against *A. baumannii*. We have added lines 389-395 to clarify this rationale in the manuscript.
**2.** Along with the Identification of New Drug Targets, new chemicals could be proposed that could work on these targets. Otherwise there is no point of indentifying targets.

**Response:** Thank you for your thoughtful comment. We appreciate the importance of linking drug target identification with potential therapeutic compounds.

In this study, our primary goal was to identify novel drug targets in *Acinetobacter baumannii* through bioinformatics tools, such as molecular docking and virtual screening. While proposing new chemical compounds is a natural next step in drug discovery, it was not the focus of this work. Drug discovery requires significant resources and specialized databases, which are beyond the scope of our study.

The key objective here was to identify reliable, novel targets, as this foundational step is critical for effective therapeutic development. Validating these targets through wet lab experiments will provide the necessary biological context before moving on to drug discovery simulations.

We plan to build upon this work in future studies by exploring drug candidates for these targets once they are validated experimentally. For now, we believe this approach provides significant value by laying the groundwork for future drug development.

**3.** Previous studies utilizing the simialr approch should be discussed along with other reported vaccines in A. baumannii strains.

**Response:** In response to the reviewer's comment regarding including previous studies, we have expanded the discussion to highlight relevant research on similar approaches and vaccine candidates for *A. baumannii* in lines 511-545.
