# Supplementary material for: Development of a multi-epitope vaccine against Acinetobacter baumannii: A comprehensive approach to combating antimicrobial resistance
Source: PLoS One. 2025 Mar 10;20(3):e0319191. doi: 10.1371/journal.pone.0319191 (PMC11892874; doi:10.1371/journal.pone.0319191)
Supplement: Attachment — Submitted filename: Response 3.docx [file pone.0319191.s006.docx]

**Dear reviewer,**

**Thank you for your constructive comments; all your comments applied to the manuscript have been highlighxted in yellow.**

1. Reviewer #3: How the multi-epitope vaccine will be synthesised and work in the body? The simulation study should be performed in triplicate and the result should be presented as the average of three.

**Response:** Thank you for your valuable comments and suggestions. We have carefully addressed your concerns as follows:

1. **Synthesis and Function of the Multi-Epitope Vaccine (MEV):** To address the query regarding how the multi-epitope vaccine will be synthesized and work in the body, we have detailed the simulation methodology in the manuscript (lines 288-293, Section 2.3.7). The immune simulation was performed using the C-ImmSim server, which characterizes the profile of the stimulated immune response by the MEV. Key simulation parameters, including the dose (1000 vaccine molecules), random seed (12,345), simulation volume (10 µl), and simulation steps (100), were specified to replicate conditions mimicking the human immune response.
2. **Simulation Study Replication and Results Presentation:** We have performed the simulation study in triplicate, as suggested, to ensure the robustness and reproducibility of the findings. The results are presented as the average of three independent simulations and have been incorporated into the results section (lines 419-446). To provide visual representation, we have also included Figure 6 in the manuscript, which illustrates the immune response trends.
3. **Discussion Enhancement:** To better explain the implications of the immune simulation findings and their relevance to MEV functionality in the body, we have expanded the discussion (lines 584-613). This section now contextualizes the simulation results and discusses how the MEV stimulates specific immune pathways and potential protective immunity in humans.

We hope these revisions satisfactorily address your comments and enhance the quality of the manuscript.

2. two different studies were merged together in the study, that does not fit.

**Response:** Thank you for your insightful comment regarding the merging of two different studies, which you indicated does not fit within the scope of our work. After carefully considering your feedback, we have made substantial revisions to the manuscript to ensure it aligns with a cohesive research focus.

1. **Focus on Reverse Vaccinology:**
   Based on your comment, we have revised the manuscript to exclusively emphasize reverse vaccinology and its application in the identification and development of a multi-epitope vaccine. All sections related to the identification of drug targets have been removed to streamline the study and avoid confusion. This refinement ensures that the manuscript adheres to a singular, well-defined research objective, enhancing its clarity and relevance.
2. **Abstract Revision:**
   The abstract has been thoroughly revised to reflect the focused scope of the study, emphasizing only the methodologies and findings pertinent to reverse vaccinology. By doing so, we have aligned the abstract with the updated content of the manuscript, ensuring consistency and clarity in the presentation of our research.
3. **Benefits of the Revision:**
   By narrowing the scope to reverse vaccinology, the manuscript now provides a more detailed and robust discussion of the vaccine design process, ensuring a clearer narrative and stronger alignment with the primary aim of the study. This streamlined approach enhances the scientific value of the work while addressing your concern about merging two disparate studies.

3. How the target protein were identified and what kind of drug could be proposed for that.

**Response:** Thank you for your thoughtful question regarding identifying target proteins and the potential drugs that could be proposed. In response to prior reviewer feedback, we have refined the scope of our study to focus exclusively on vaccine development using reverse vaccinology. As a result, all sections related to drug target identification and associated drug proposals have been removed from the manuscript to maintain clarity and consistency with the study's primary objective.

The target proteins discussed in the manuscript were identified specifically for their potential as vaccine antigens. This process involved computational techniques such as [e.g., antigenicity prediction, epitope mapping, immunogenicity scoring] to select proteins capable of inducing a strong immune response. These proteins were not analyzed in the context of drug targeting or therapeutic intervention.

We appreciate your insightful comment and recognize the importance of exploring target proteins for drug discovery. While this aspect is beyond the scope of the current study, it could be a valuable avenue for future research.

4. Perform the simulation of the proposed vaccine against human MHC.

**Response:** Thank you for your valuable suggestion regarding performing the simulation of the proposed vaccine against human MHC. We sincerely appreciate your input, which has helped us to further refine and evaluate our study.

We would like to inform you that we have conducted immune simulations to characterize the immune response elicited by the proposed vaccine. The details and results of these simulations have been incorporated into the manuscript, demonstrating the vaccine's potential efficacy in stimulating an immune response.

However, regarding your suggestion to perform simulations specifically against human MHC, we regret to inform you that this aspect was not executable within the scope of this study due to the current limitations in computational resources and expertise within our team. Despite this limitation, we have ensured that the immune simulation results align with the study's goals and provide meaningful insights into the vaccine's performance.

We acknowledge the importance of MHC-specific simulations and agree that they could further enhance the study. We plan to explore this aspect in future research by collaborating with experts in the field or utilizing more advanced tools.
